# Meteorology and geography, more than biological traits, drive variation in frog phenology across decades

**DOI:** 10.1002/ecy.70394

**Published:** 2026-05-05

**Authors:** David H. Klinges, L. Kealoha Freidenburg, Adriana D. Rubinstein, David K. Skelly

**Affiliations:** ^1^ School of the Environment Yale University New Haven Connecticut USA

**Keywords:** amphibian, biophysics, climate, endogenous, exogenous, microclimate, phenology

## Abstract

The fate of a species is a function of interacting environmental and biological processes. Disentangling the roles and interactions of such processes can elucidate the breadth of possible responses to global change, for instance, the potential for phenotypic plasticity or trait evolution to rescue populations from climate change. We explored how environmental and biological factors influenced the timing of emergence post‐hibernation, and subsequent oviposition, of the temperate aquatic amphibian, *Rana sylvatica*. We evaluated how frog phenology changed for 64 populations over 25 years, pairing these observations with 45 years of mechanistic and machine learning simulations of microclimate and frog physiology. Adult frog oviposition dates varied between day‐of‐year 74 and 135, and on average advanced marginally by 1.6 days per decade. Coupled mechanistic models predicted frog emergence date with a median absolute error of 6.9 days (RMSE: 8.95 days). Sensitivity analyses of the mechanistic simulations demonstrated the importance of vegetation structure and meteorology, and their interactions, for driving variation in emergence dates, while frog behavior played a moderate role. Modeled variation in morphological and physiological traits had little effect on predicted phenological variation, even when trait space was unrealistically inflated. Our study suggests that for this system, interpopulation variability in phenology may be driven more by exogenous factors (the environment), and to a lesser extent behavior, rather than endogenous traits of morphology and physiology, the latter of which may provide little capacity to respond to changing climates over time. Our approach suggests that pairing phenological observations with generalizable mechanistic models can offer an effective platform to understand and predict responses to global change.

## INTRODUCTION

**FIGURE 1:**
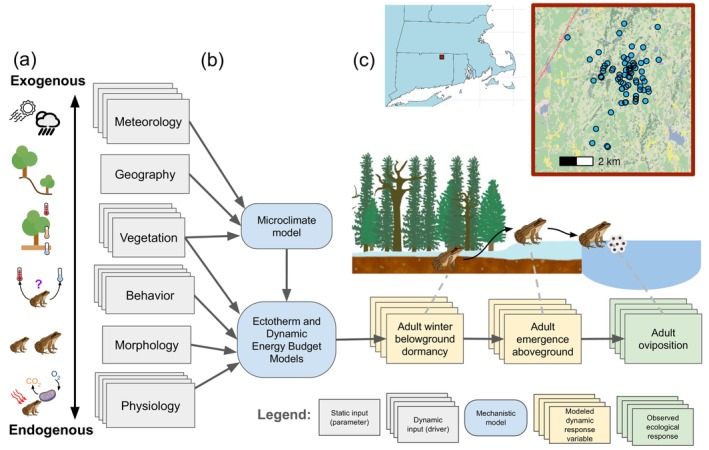
(a) From the perspective of a single species, ecological variables can vary from environmental conditions that are fully external to the organism (exogenous) to biological traits that are fully intrinsic to the organism (endogenous). (b) We integrated empirical data with mechanistic models to explore how the roles of exogenous and endogenous variables drive variation in the timing of post‐hibernation emergence of wood frogs (*Rana sylvatica*). Wood frogs hibernate below leaf litter and snow cover, and based on environmental and physiological cues, emerge from belowground and navigate to a nearby (and frequently their natal) pond to breed and deposit eggs. (c) We leveraged 45 years of observations for populations associated with 64 ponds in northeastern Connecticut. Illustrations/icons created by David H. Klinges.

The ecology of organisms arises from the interactions among multiple drivers, some of which can be categorized into climatological, geographical, and biological processes (Wainwright & Reilly, 1994). From the perspective of a single species, drivers of phenology, development, reproduction, and other processes can be considered as falling along a gradient from exogenous to endogenous with respect to that species (Figure 1). Exogenous drivers are those environmental and ecological conditions that are external to the organism, such as ambient meteorology, topography, and human modification of habitat, which serve as the context within which biology plays out. Endogenous drivers, on the other hand, are those biological traits and conditions that are intrinsic to a species, including physiology, behavior, and dispersal, which arise from genetic variation, demography, and culture (Baecher et al., 2023; Bolker, 2003; Porter et al., 2023).

The interactions between exogenous and endogenous factors determine the rate and magnitude of ecological processes. Yet, the relative importance of exogenous versus endogenous drivers for shaping variation in an ecological process may determine the resilience of a given population to environmental change (Urban et al., 2013). If endogenous factors explain a large amount of variation in a population's response to change, this may be due to high phenotypic plasticity, standing genetic variation, or behavioral adjustment—all of which may entail broader biological capacity to withstand adverse or variable conditions (Huey et al., 2012; Sunday et al., 2014). Conversely, if endogenous factors explain little variation in a population's response to change, relative to exogenous factors, this suggests a limited biological toolkit by which the organism can adapt to environmental change—the organism is more at the whim of its environment, possibly entailing a higher likelihood for an individual or population to succumb to perturbations. From a practical standpoint, understanding the importance of exogenous versus endogenous factors can also guide how well researchers can model or predict an ecological system. Relative to biological processes, which are frequently hard to measure or model at scale, many (but not all) meteorological and environmental processes are well represented by both mechanistic models and spatial gridded data (Randin et al., 2020). An ecological response for which exogenous factors are more important than endogenous ones therefore may be understood and predicted at broad scales even when trait‐based measurements cannot be scaled, if the important exogenous factors are identified.

Phenology, or the relative timing of life events for an organism, serves as one of the most important processes through which biological traits and the environment interact, including via a species’ response to contemporary climate change (Cleland et al., 2007). To maintain tolerance to dynamic climates, species must either adapt, shift their life history in time (phenology), and/or shift in space (Parmesan, 2006). Recent work has shown that phenological shifts in response to climate change are idiosyncratic and often linked to both environmental factors and resource‐tracking cues (Lang et al., 2024; Neate‐Clegg et al., 2024), although few organisms shift phenology fast enough to keep up with climate warming (Loughnan et al., 2024; Piao et al., 2019). Understanding the pace of phenological shifts, and the exogenous and endogenous drivers of any such phenological shifts, is important for species‐specific conservation (Timm et al., 2007), natural resource management (Willems et al., 2021), and building generalized theory (Primack et al., 2023).

Mechanistic niche models offer a particularly useful platform by which to evaluate the relative importance of exogenous versus endogenous factors for shaping ecological responses including phenology (Kearney & Porter, 2009), as such models explicitly represent the interactions of environmental and biological variables across the gradient of exogenous to endogenous (Figure 1). For example, meteorology (extrinsic for and agnostic to all species, and therefore fully exogenous) shapes local microclimates that organisms experience and from which mobile animals can behaviorally select (where exogenous and endogenous factors meet). Then, an organism's morphology and physiology (all intrinsic to individual organisms, and therefore endogenous) are examples of traits that are subject to selection, providing the biological substrate by which evolution operates. All such environmental and biological variables can be explicitly represented through time via mechanistic niche models (Briscoe et al., 2023). Given their grounding in theory, mechanistic models in particular allow one to leverage proposed mathematical relationships of exogenous and endogenous factors to evaluate how sensitive an ecological response is to different factors (Ma et al., 2023).

Wood frogs (*Rana sylvatica = Lithobates sylvaticus*) are a widespread pond‐breeding amphibian occurring across much of North America (Dodd, 2013; Lee‐Yaw et al., 2008). During winter, adult frogs hibernate belowground, and based on a combination of environmental and biological cues, which are not fully known, they emerge and navigate to nearby vernal ponds to reproduce (Arietta et al., 2020). Selection of hibernaculum microhabitats by wood frogs, and subsequent emergence phenology, serve as important stages of the animal's life history, in some cases dramatically impacting adult survival rates (Fitzpatrick et al., 2020; O'Connor & Rittenhouse, 2016). Wood frog dispersal is spatially limited (Bellis, 1965; Berven, 1982; Skelly et al., 1999), and as ectotherms their physiology and behavior are sensitive to their local environment and microclimate (Angilletta Jr., 2009)—both reasons why organismal biophysiology models are useful tools for exploring exogenous and endogenous drivers of their responses to global change (Briscoe et al., 2023).

We evaluated the relative importance of exogenous and endogenous factors for the phenological response of the wood frog to climate change. Combining 25 years of observations of wood frog phenology with mechanistic simulations of microclimate and animal physiology, metabolism, and behavior, we ask: How do environmental context and amphibian biology separately, and jointly, shape variation in the timing of adult frog emergence at the end of winter? Understanding the drivers of winter emergence phenology can serve to advance understanding of intra‐ and intergenerational responses to global change for a widespread vertebrate species. We examined mechanistic model accuracy at capturing phenological observations, relative to the accuracy of data‐driven machine learning, and performed simulation experiments to evaluate whether wood frog phenology is more sensitive to exogenous or endogenous factors. We then contextualize how our approach here, combining observations, trait values, and mechanistic niche models, aids broader understanding of the capacity by which organisms can use behavior and biology to mitigate impacts of climate variability.

## METHODS

### Natural history and phenological data collection

Wood frogs are one of the few vertebrates capable of withstanding internal ice formation, given their capacity to accumulate glucose in their bloodstream to serve as a cryoprotectant (Storey & Storey, 1984). Adults overwinter by burying below litter into the topsoil, typically 2–5 cm deep, in forested or otherwise vegetated habitats, selecting for microhabitats that are thermally buffered throughout winter (to avoid exposure to metabolically taxing freeze–thaw cycles) yet exposed enough to solar radiation so as to warm and melt early in spring (Groff et al., 2016; O'Connor & Rittenhouse, 2016; Sinclair et al., 2013). This also entails that wood frogs are generally the first active amphibians in spring throughout their range, well before all snow and ice have melted across a landscape (Dodd, 2013). Upon emergence, adult males move to a temporary pond within close vicinity (often the same pond at which the individual was born) to chorus en masse and attract females, after which reproduction and oviposition occurs in the shallow edges of the pond (Bellis, 1965; Berven, 1982).

We monitored wood frog phenology from 2000 to 2024 for 64 nonpermanent wetlands (hereafter ponds) in northeastern Connecticut within the 3213‐ha Yale Myers Forest (Figure 1), with additional modeling for all ponds from 1979 to 2024. While the focus of this study was upon adult frog emergence post‐hibernation, monitoring the timing of emergence at scale (across years and many populations) presents an extreme challenge, given the diversity of hibernacula microsites surrounding any given pond. Instead, the timing of oviposition (egg deposition within ponds) serves as a more logistically feasible measurement of phenology. Therefore, we used records of wood frog oviposition from 2000 to 2024, as described in Arietta et al. (2020). In brief, two observers searched each pond for egg masses, no less than once per week after early March (the earliest recorded wood frog oviposition in the area). If eggs were found, the observers independently counted masses, and then averaged the estimates (for some surveys with one observer, egg masses were counted twice and averaged). As breeding commences shortly after emergence from hibernation, dates of oviposition served as an approximate, although not exact, indicator of per‐pond average adult emergence dates.

### Modeling air and soil microclimates

We used the mechanistic microclimate model of NicheMapR (Kearney & Porter, 2017) to simulate the thermal, hydric, and radiative conditions that adult frogs experience while in hibernacula belowground and active aboveground. This model represents heat, radiative and hydric exchange across a set of vertical nodes (above‐ and belowground) at hourly timesteps for high and low radiative shading, therefore representing the proximal conditions below vegetation and subject to topography as are experienced by most terrestrial, arboreal, fossorial, and aquatic organisms. The microclimate model was run via the *micro_usa()* function of NicheMapR and parameterized with observations and estimates of meteorology, vegetation, topography, and pedology for each pond (Appendix S1: Table S1). In brief, this included: gridMET daily surface meteorology at approximately 4‐km spatial resolution (Abatzoglou, 2013), a 0.8‐m digital elevation model of Connecticut (CT ECO, 2016); vegetation properties such as leaf area index and canopy height as estimated for mixed forest by *microclimc* (Maclean & Klinges, 2021); and soil properties using the *pedotransfer()* function of NicheMapR following methods by Enriquez‐Urzelai et al. (2019); full parameterization of the model is available via Zenodo (Klinges & Skelly, 2026). To represent shading levels for microclimate simulations at each pond, following Billet et al. (2024), we used time‐constant percent forest cover estimates within a 200‐m radius around each pond, derived from 30‐m resolution imagery from the 2019 US Geological Survey National Land Cover Database (Homer et al., 2012). We also performed modeling with empirical estimates of minimum and maximum canopy cover from hemispherical photographs (Arietta et al., 2020). Model predictions were more accurate using 200‐m radius forest cover estimates, and therefore these were used in reported results.

### Modeling frog activity and phenology

We used the mechanistic ectotherm and dynamic equilibrium energy (DEB) models of NicheMapR (Kearney & Porter, 2020) to then simulate adult frog activity subject to environmental exposure (Figure 1). These models in tandem represent an organism as an open thermodynamic system exchanging energy with its environment. State‐specific budgets for heat and water interact with metabolism (feeding, maintenance, and development) to determine growth and life history, modulated by an organism's activity through foraging and thermoregulation. However, important processes are therefore neglected, including dispersal, competition, and predation. As with the microclimate model, these animal models represent vertical conditions at a point with high‐shading and low‐shading options, rather than a spatial grid of conditions. As such, thermoregulation entails (1) vertical movement (e.g., burrowing deeper), (2) posture adjustments (relative to the modeled direction of the sun's rays and to change amount of contact with the soil substrate), and (3) basking or shade‐seeking behavior, all to avoid lethal extremes and to optimize exposure to preferred conditions (Kearney & Porter, 2020). We used environmental conditions as estimated by the NicheMapR microclimate model to drive the ectotherm and DEB models, with empirical records or estimates of wood frog morphology, physiology, and behavior provided as model parameters (Appendix S1: Table S2). Metabolic traits for the DEB model were drawn from published records for the Siberian wood frog (*Rana amurensis*), which has similar ecology and physiology (including freeze tolerance) to *R. sylvatica* (Shekhovtsov et al., 2020).

We modeled wood frog activity from 1979 to 2024 for each pond in Yale Myers Forest separately, discarding the first year of predictions as burn‐in. Each simulation represented a single frog at a single location adjacent to each pond. While locations of adult wood frog hibernacula may be over 500 m away from their aquatic breeding site, most individuals remain within 100 m of their natal pond (Bellis, 1965; Groff et al., 2017), justifying our use of environmental data describing conditions surrounding ponds. As with microclimate modeling, wood frog development and activity were modeled hourly.

Meteorological and environmental conditions determined the range of microclimates available to an adult frog, which was initialized belowground. Based on these microclimatic conditions, the simulated frog would attempt to maintain the most desirable energetic status. We constrained the frog to remain belowground, representing the dormant state of adult frogs in winter, until a set of conditions were met:

1. The simulation advanced beyond the 65th day of the year (earlier than any observed frog activity in Connecticut, Arietta et al., 2020).
2. Wood frog body temperature was above −0.16°C for three consecutive days (corresponding to the inflection temperature at which frog thawing begins, Sinclair et al., 2013).
3. There was no snow for at least five consecutive days (Fitzpatrick et al., 2019).

When all of these criteria were met, frogs were then permitted to emerge, which would occur if aboveground conditions were otherwise physiologically desirable for activity (Appendix S1: Figure S1).

### Machine learning predictions

To evaluate the performance of the mechanistic predictions against a data‐driven model, we also trained a random forest model to predict the first day of wood frog oviposition. This random forest was fitted directly to the observed dates of frog egg records, while NicheMapR instead predicted the timing of adult frog emergence. Furthermore, by fitting this random forest model as described in Arietta et al. (2020), we were able to directly compare our predictions to this prior study of wood frog phenology. In brief, we trained the model using pond‐wise covariates of latitude, elevation, aspect, and canopy cover metrics, along with site‐wide daily average temperature, precipitation, and snow water equivalent from DayMet (Thornton et al., 2021) between day‐of‐year 0 and 120 (1 Jan–29 April). We grew our random forest from 1000 regression trees. We then used the model fit to all observations to hindcast oviposition dates between 1980 and 1999, and predicted in‐sample for each pond from 2000 to 2024.

As a null model against which to evaluate mechanistic and machine learning predictions, we also simulated frog biophysiology in response to microclimatological day‐of‐year normals. This aids interpretation of the value of exogenous drivers, which are held constant across years in this null model while dynamic in our mechanistic and machine learning models. For each pond, we averaged microclimate time series across years from 1979 to 2024 to the average conditions for each day‐of‐year. We then concatenated 46 replicates of such microclimatological normals to derive 46 years of identical annual conditions. We then drove the NicheMapR ectotherm model using these microclimatological normals, again discarding 1979 as burn‐in.

### Validation

We validated the mechanistic predictions of microclimatic air temperature, total precipitation, snowfall, snow depth using 4 years of meteorological observations from a weather station within Yale Myers Forest, and from 7 years of observations from a NOAA Global Historical Climatology Network weather station in Eastford, CT (41.899067 N, 72.120719 W). We used these observations to validate microclimate simulations for a relatively open canopy (0% minimum shade, 10% maximum shade), corresponding to the locations of both weather stations. We calculated the root mean square error (RMSE) of climate predictions from measurements, calculated at hourly, daily (means/minima/maxima), and monthly (means/minima/maxima) resolutions. Snow predictions were only validated at daily and monthly resolutions, as measurements were made daily.

We validated predicted dates of wood frog emergence with observations of frog egg masses between 2000 and 2024. While the NicheMapR ectotherm model does have modularity to predict oviposition, built‐in parameters that determine timing of oviposition (e.g., oviposition timing based on daylength) do not represent wood frog phenology, and through exploration of NicheMapR oviposition predictions, we determined the model was unsuitable for capturing wood frog oviposition observations directly. Preliminary investigation indicated that the NicheMapR model was sensitive to the narrow range of elevation expressed across ponds, which introduced bias in predicted emergence rates (Appendix S1: Figure S3); therefore for validation, we set elevations of all ponds equal to the mean pond elevation (236 m ASL). We calculated the RMSE and absolute error of the predicted day of frog emergence from the day of observed egg masses per pond per year. For years when multiple surveys were recorded for the same pond, we averaged survey dates weighted by the number of new egg masses recorded in subsequent surveys.

Drivers of the timing of frog oviposition (used for validation) may differ from drivers of emergence (what was mechanistically modeled). For instance, this may include the severity of drought conditions, as successful reproduction and oviposition entail rain events and standing water in vernal pools (Berven, 1982). Thus, to explore error in mechanistic emergence predictions, we fit a generalized linear model (GLM) with error in mechanistic predictions as the response (modeled with a Gaussian link) and several covariates: early‐spring drought severity, per‐pond abundance of egg masses, pond average canopy cover, and pond area. For estimating early‐spring drought, we extracted time series of the Palmer Drought Severity Index (PDSI, Palmer, 1965) from gridMET daily gridded meteorological predictions (Abatzoglou, 2013) for the grid cell of central Yale Myers Forest and averaged PDSI values for the months of March and April for each year. Prior to fitting the GLM, all covariates were scaled between 0 and 1 to standardize the contributions of each parameter.

### Phenological trends over time

To explore phenological trends in oviposition across 25 years of observations, per Arietta et al. (2020), we quantified the change in per‐pond phenology via a linear mixed‐effects model fit to oviposition observations, predicting oviposition date by year, including the pond of observation as a random intercept both with and without allowing the slopes of the relationship with year to vary among ponds as a random effect. For all linear models, we calculated 95% confidence intervals (CIs) for coefficient estimates with 1000 bootstrap iterations.

### Sensitivity analyses

To measure the importance of exogenous (meteorological, geographic) and endogenous (behavioral, physiological, morphological) processes for wood frog phenology, we conducted one‐at‐a‐time sensitivity analyses, in which we estimated the sensitivity of predicted date of frog emergence to variation in each of a set of parameters inputted to the NicheMapR microclimate and animal models (Appendix S1: Table S3). Minimum and maximum values of each parameter were defined based on previously published estimates, or the range of empirical values observed across all ponds at our study site, with an additional parameter value at the mean between minimum and maximum (totaling three values per parameter). We ran a set of simulations with the NicheMapR microclimate and ectotherm models from 1979 to 2024 (discarding 1979 as burn‐in). For each simulation, we changed the value of a single parameter while all others were held constant at their mean value. Within each 45‐year simulation, we summarized emergence dates to their interannual mean. We then reported the sensitivity of such mean emergence dates to each parameter based on the difference in the lowest and highest mean emergence dates predicted from simulations in which that parameter was allowed to vary. This method allowed for nonlinear effects of the parameter on emergence dates, as opposed to reporting sensitivity based on the difference in emergence dates between the minimum and maximum values of the parameter (Holmquist et al., 2018). As the roles of animal traits may differ depending on whether the NicheMapR dynamic energy budget (DEB) model is implemented or not, we ran sensitivity analyses both with and without the DEB model activated. We also reconducted the sensitivity analyses with inflated ranges of biological trait values, to represent broader phenotypes and/or plasticity. We additionally performed sensitivity analyses of the minimum and maximum microclimatic (below‐canopy) air and soil temperatures, but only to the meteorological and geographical parameters (as frog biological parameters were irrelevant to microclimate).

### Software

All analyses were conducted in R v4.2 (R Core Team, 2024). A list of critical R packages and their citations is provided in the Supporting Information. We thank the efforts of many for producing open‐source software to advance research efforts. Data and code from this study are available via Zenodo (Klinges & Skelly, 2026).

## RESULTS

### Validation

**FIGURE 2:**
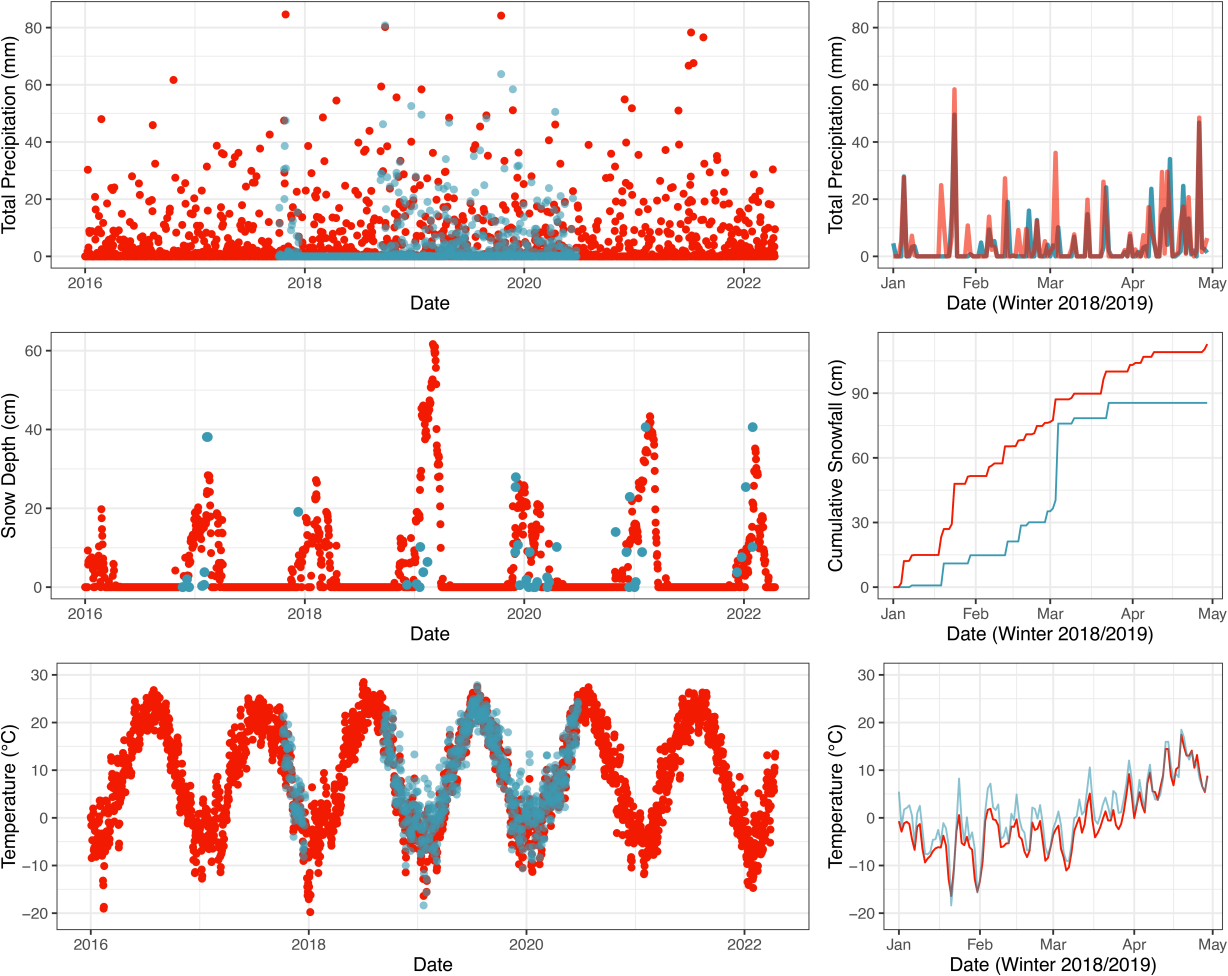
Sample time series for mechanistic model‐predicted micrometeorology in Yale Myers Forest, for a broader temporal duration (left panels) and just for the winter of 2018–2019 (right panels). Red lines/points indicate model predictions, with measurements in blue. GridMet‐predicted precipitation and NicheMapR‐predicted snowfall, snow depth, and temperature all captured empirical variation with reasonable accuracy.

GridMet meteorology and the NicheMapR microclimate model were both reasonably accurate at representing empirical conditions as measured by local weather stations (Figure 2). GridMet predicted daily total precipitation with a RMSE of 7.21 mm and mean absolute error (MAE) of 2.97 mm (Appendix S1: Table S4). When summed to daily totals, NicheMapR‐predicted snowfall had RMSE of 4.75 cm and MAE of 1.52 cm, and predicted snow depth had RMSE of 14.9 cm and MAE of 11.69 cm (Appendix S1: Table S5). NicheMapR‐predicted microclimatic hourly air temperatures had RMSE of 4.00°C and MAE of 3.11°C (Appendix S1: Table S6).

**FIGURE 3:**
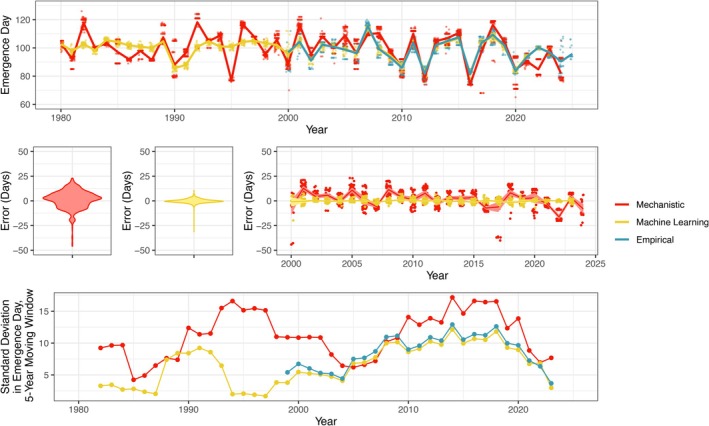
Top: Validation of mechanistic (NicheMapR) and machine learning (random forest) predictions of frog phenology using observations from 2000 to 2024. While mechanistic predictions were of post‐hibernation emergence, machine learning and validation data were of frog oviposition, which occurs shortly after emergence. Middle: Mechanistic predictions had a median absolute error of 6.9 days, while machine learning predictions were more accurate, with a median absolute error of 2.38 days. Bottom: Yet, when evaluating interannual variation in predictions (i.e., the SD in predictions across 5‐year moving windows), mechanistic predictions generally had high variation across time, while machine learning had reduced variation for out‐of‐sample prediction (years 1979–1999) relative to in‐sample prediction (2000 onwards), suggesting possible regression towards the mean by the random forest.

NicheMapR predicted the day‐of‐year of adult frog emergence with RMSE of 8.95 days and MAE of 6.9 days (Figure 3). As indicated by a GLM, error in NicheMapR emergence predictions correlated with several predictors: the severity of drought in early spring months (ꞵ = −0.54, SE = 0.24, 95% CI = −1.02, −0.07) and the mean egg count of a pond (ꞵ = −0.80, SE = 0.24, 95% CI = −1.27, −0.32) were both important predictors of NicheMapR error. Therefore, removing years that experienced severe drought (PDSI below −2.5) entailed slightly higher prediction accuracy (RMSE of 8.88 days), as did subsetting to just those ponds and years falling within the upper 50% quantile of recorded egg mass counts (30 egg masses; RMSE of 7.58 days). The coefficient estimates of the pond canopy cover (ꞵ = 0.37, SE = 0.27, 95% CI = −0.16, 0.90) and pond area (ꞵ = −0.33, SE = 0.27, 95% CI = −0.86, 0.20) did significantly deviate from zero.

Fivefold cross‐validation showed that, the random forest had higher predictive accuracy than the mechanistic models (RMSE = 3.04 days; MAE = 2.38 days; Figure 3). Yet, interannual variation in random forest predictions was substantially reduced for out‐of‐sample hindcasting before year 2000 (SD = 4.47) relative to in‐sample prediction (SD = 8.30), the latter of which reflected empirical variation (SD = 8.94; Figure 3), suggesting possible out‐of‐sample regression towards the mean (Gareth et al., 2013). NicheMapR, conversely, had comparable interannual variation both before (SD = 10.8) and after (SD = 11.6) year 2000. Both the random forest and NicheMapR models substantially outperformed a null model using microclimatological normals (RMSE = 28.69 days; MAE = 27.08 days).

### Phenological trends over time

From observations, oviposition dates ranged from day‐of‐year 74 to day‐of‐year 135, with a median of day‐of‐year 99. The linear mixed‐effects model of oviposition date over time predicted an average trend of phenological advance per decade averaged across all 64 ponds (ꞵ = −0.16, SE = 0.039, 95% CI = −0.24, −0.08, Appendix S1: Figure S4). Fitting a mixed model allowing slopes to vary by pond (AIC = 9800.5) did not increase parsimony relative to a model without pond‐specific slopes (AIC = 9796.5). Subsetting oviposition data to 2000–2019 supported findings of phenological delay as found by Arietta et al. (2020) (ꞵ = 0.084, SE = 0.053, 95% CI = −0.010, 0.18), but the additional 5 years of data (2020–2024) switched the estimated direction of phenological shift.

### Sensitivity analysis

**FIGURE 4:**
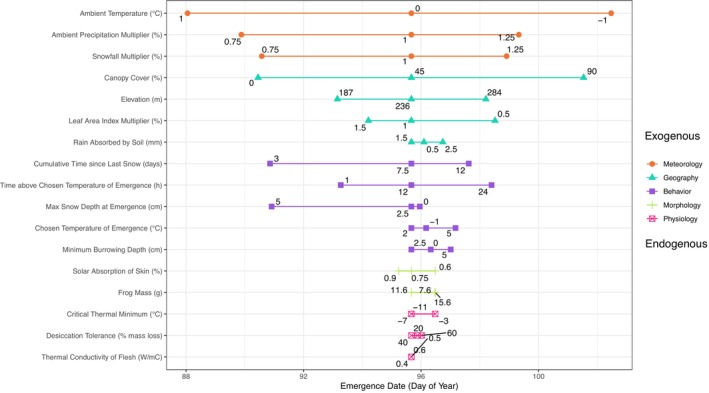
Sensitivity of adult frog post‐hibernation emergence to a range of parameters ranging from fully exogenous to fully endogenous. Each point indicates the average emergence day‐of‐year for a single frog across a 45‐year simulation (1980–2024). Quantitative labels on each colored segment indicate the value of the input parameter for that simulation; all other parameters were held at their average value (i.e., label of middle point for each segment). Increasing width of a segment entails stronger importance of a parameter for driving variation in frog emergence (parameters are ordered by category and importance). Exogenous parameters generally were more important than endogenous parameters for driving variation in emergence date. See Appendix S1: Table S3 for an explanation of all parameters.

Emergence phenology as predicted by the mechanistic NicheMapR model was generally more sensitive to exogenous parameters (meteorology, geography) than endogenous parameters (physiology, morphology, behavior). With the DEB model turned off, meteorological parameters explained 42% of all variation in emergence date, geography explained 28%, behavior 25.8%, morphology 2.7%, and physiology 1.5% (Figure 4). With the DEB model on, exogenous parameters still explained 63.3% of all variation in emergence (Appendix S1: Figure S5); variation in emergence explained by geographic, physiological, and behavioral parameters increased by 13.7%, 5.7%, and 2.4%, respectively, while meteorology and morphology explained 20.4% and 1.4% less variation, respectively. When the ranges of trait values were inflated, however, endogenous factors explained 50.2% of all variation, yet this was because a single quasi‐behavioral parameter—the number of consecutive hours above the chosen temperature of emergence—explained 31.7% of all variation (Appendix S1: Figure S6). Air and soil microclimatic temperatures were generally more sensitive to canopy cover than meteorological or other geographic parameters (Appendix S1: Figures S7 and S8).

## DISCUSSION

### Exogenous factors, more than endogenous factors, drive frog phenology

While ecology arises from the interactions of organisms with their environment, disentangling the roles of exogenous and endogenous factors helps explain the extent to which a given species or population may exhibit biological resilience to disturbances. Pairing mechanistic models with long‐term observations, we evaluated how exogenous factors (meteorology, geography) and endogenous factors (behavior, morphology, physiology) drive the phenology of adult wood frogs emerging from winter hibernation to reproduce. Such modeling captured observed phenology with high accuracy (Figure 3). Furthermore, via a simulation experiment and sensitivity analysis, we found that the timing of frog emergence was more sensitive to exogenous factors than endogenous ones (Figure 4).

Meteorological parameters (ambient temperature, total precipitation, and snowfall) accounted for 42% (32.2 days) of all variation in emergence day, while geographic parameters (canopy cover, leaf area index, and elevation) accounted for 28% (21.5 days) of all variation in emergence day. For instance, a 2°C increase in ambient temperature yielded an average shift of frog emergence earlier by 14.4 days, while increasing canopy cover (from a 0% shaded to a 90% shaded pond) delayed frog emergence on average by 11.1 days. Conversely, adjusting morphological and physiological trait values yielded almost no change in phenology; for instance, doubling frog mass from 7.6 to 15.6 g shifted average emergence by only 0.8 days. The poor performance of our null model—which was restricted to temporally averaged climatology, yet still allowed for variable behavior of simulated frogs—lends further evidence of the importance of environmental drivers and representing them dynamically for adequate predictions.

**FIGURE 5:**
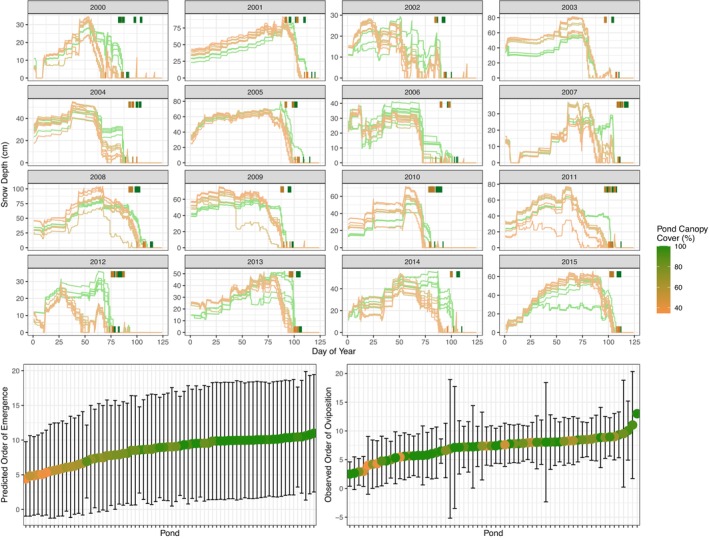
A proposed pathway by which meteorology and geography interact to influence wood frog emergence. (Top) As shown here for a subset of studied years (2000–2015), snow depth generally increased through the early winter and melted in spring, with accumulation and melting rates dependent on the amount of canopy cover (which intercepts snowfall). Vertical tick marks indicate per‐pond emergence dates as observed (top ticks) and predicted (bottom ticks). (Bottom left) This entails that the mechanistic models predicted the order of emergence dates across ponds to consistently correspond to the amount of canopy cover. (Bottom right) Empirical order, however, did not demonstrate a clear signal of canopy cover, indicating the importance of other unmodeled processes.

Microgeographical variation in forest structure surrounding ponds situated just a few hundred meters apart yielded distinct microclimatic regimes (Appendix S1: Figures S7 and S8) that caused frog emergence to decouple across populations (Figure 3). These findings corroborate a recent study that found no spatial synchrony in frog oviposition phenology across the same set of metapopulations (Rowland et al., 2022). Mechanistic modeling further suggested a clear and intuitive pathway of how meteorology and local geography jointly shape wood frog emergence. Snow accumulates and melts throughout the winter, with more vegetated ponds receiving less peak snowfall given canopy interception, but longer persistence of snow into the spring, relative to more open ponds (Figure 5). With such early snow melt, mechanistic models predicted earlier frog emergence at open ponds relative to vegetated ponds (Figure 5).

Prior work has suggested the importance of not just forest vegetation structure for shaping anuran winter ecology and tadpole development (Skelly et al., 2002), but also that finer scale microhabitat features drive wood frog hibernaculum selection and hibernation survival by determining local snow depth. Deeper snow cover insulates soil hibernacula from thermal variability, reducing exposure to lethally extreme cold temperatures (O'Connor & Rittenhouse, 2016). Wood frogs have been observed to select hibernacula next to rocks and woody debris, which accumulate windthrown snow (Groff et al., 2016). Similarly, greater leaf litter depth or the presence of moss both decreases thermal variability and increases moisture content, reducing the risk of desiccation (Churchill & Storey, 1993). These microhabitat features were represented implicitly in the sensitivity analysis via tuning of the amount of rain absorbed by soil and adding a snowfall multiplier, the latter of which strongly influenced emergence date. Explicit representation of such microscale drivers of phenology may help evaluate the importance of meter‐scale heterogeneity relative to landscape‐scale heterogeneity.

The role of snowfall and snow cover in shaping frog phenology further illustrates the importance of standing snow for winter ecology (Slatyer et al., 2022). The subnivium—the seasonal microclimate refugium at the interface between snow and the soil surface—serves as a critical resource for winter survival and development for animals, plants, and microbes across systems (Pauli et al., 2013). Historic measurements and modeled forecasts indicate decreased snow cover and duration of the snow season in the Northern Hemisphere (Brown & Robinson, 2011; Choi et al., 2010). Winter recreation and land management, including snowmobiling, skiing, and silviculture, may further erode or homogenize the subnivium (Zuckerberg & Pauli, 2018). Pairing process‐based meteorological and niche models, as we did here, facilitates development of hypothesized causal relationships between decreasing snow cover and physiological ramifications, which can be further tested via snow removal experiments (Wipf & Rixen, 2010).

Of the endogenous parameters manipulated in our sensitivity analysis, behavioral traits shaped phenology more so than morphological and physiological traits. This finding corroborates recent work that has emphasized the role that behavior can play in shaping thermal tolerance, and mitigating physiological ramifications of climate change (Kearney & Porter, 2020; Muñoz et al., 2016; Sunday et al., 2014). This does not, however, entail that morphological and physiological traits are unimportant for amphibian ecology. Development rates and performance of tadpoles are closely linked to body mass and physiological condition (Stoler et al., 2015), which along with predation risk (Relyea, 2002) influences adult fitness (Berven, 1982; Skelly et al., 2002). Sex‐specific body mass also influences their predicted vulnerability to ongoing warming given mass‐specific energy expenditures during winter freeze–thaw cycles (Fitzpatrick et al., 2019). We advocate for further approaches that disentangle how exogenous versus endogenous factors influence ontogeny and full life cycle development of ectotherms such as amphibians.

### Phenological advance

Contrary to the findings of Arietta et al. (2020) for the same metapopulation, we found phenological advance of frog oviposition dates rather than delay (Appendix S1: Figure S4), despite using the same statistical method as Arietta et al. (2020) for trend estimation. The divergent findings here are due to several factors. First, our study expanded upon the time series of Arietta et al. (2020) from 20 to 25 years, possibly allowing for more informed inference and highlighting the importance of long‐term ecological studies (Reinke et al., 2019). Additionally, the years 2016 and 2017 underwent intense drought in February and March (Appendix S1: Figure S2). Such late‐winter drought can delay reproduction and oviposition, even if thermal conditions are otherwise suitable for earlier oviposition. As these were some of the last few years studied by Arietta et al. (2020), such drought may have pulled the linear trend estimate in that study towards a delay; removing the drought years by re‐fitting the model to just 2000–2014 indeed yielded an advance rather than delay (ꞵ = −0.22, SE = 0.080, 95% CI = −0.37, −0.056).

### Mechanistic and machine learning phenology predictions

While both the mechanistic NicheMapR and a random forest predicted frog phenology with comparable accuracy, the machine learning algorithm outperformed NicheMapR across several validation metrics (Figure 3). This was expected for two reasons. First, validation observations were of oviposition, while our mechanistic approach modeled adult frog emergence, which by necessity predates oviposition and is sensitive to different processes. For instance, thermal cues and the presence of snow cover may drive amphibian emergence, while rainfall or moon phases may drive movement towards ponds and oviposition (Benard & Greenwald, 2023; Parmesan, 2007; Semlitsch, 1985; Timm et al., 2007). Second, the random forest is a correlative algorithm trained on site‐specific observations to minimize error, while the mechanistic models are generalizable, process‐based approaches. Comparable performance between NicheMapR and the random forest suggests remarkably high accuracy of NicheMapR.

Yet notably, extrapolated random forest predictions may have incompletely represented interannual variation in frog phenology. The random forest yielded higher variation for in‐sample predictions (years 2000–2024), while out‐of‐sample predictions (years 1980–1999) more closely reflected the mean prediction across years (Figure 3). Such regression towards the mean is often due to penalization against outlier values during machine learning training (Gareth et al., 2013). This points to one of the trade‐offs between correlative and mechanistic approaches, with the former frequently entailing greater prediction accuracy and the latter providing better causal inference (Malmborg et al., 2024). Given these trade‐offs, global change research may best advance via use of hybrid approaches fusing both mechanistic and machine learning models (Buckley et al., 2023).

### Caveats to our approach

Our interpretation that exogenous, more than endogenous, drivers shaped wood frog phenology hinges upon our assumptions during modeling frog biology, which may have incompletely represented frog traits or overlooked important biological processes. Selected ranges of biological trait values were grounded in prior measurements so as to capture naturally occurring variation, yet broader phenotypic variation or plasticity may entail larger trait space than represented here. For instance, reconducting simulation experiments but with substantially inflated biological trait ranges entailed that endogenous factors accounted for 20.2% more variation in emergence timing relative to simulations with empirical trait ranges (Appendix S1: Figure S6). Yet, this was in large part because of the explanatory power of a single quasi‐behavioral trait: the number of consecutive hours above the threshold temperature before emergence occurs, which explained 31.7% of all variation with an inflated range of values between 1 and 96 h (relative to the prior range of 1–24 h). However, such a wide range is likely unrealistic, and for all other endogenous factors, inflating the range of trait values did not qualitatively increase their explanatory power in sensitivity analysis. This suggests that even with high genetic diversity or phenotypic plasticity, such biological traits are relatively unimportant for wood frog emergence phenology.

Additionally, the NicheMapR model assumes that an organism metabolizes and behaves in a near‐perfect manner to track its preferred conditions in response to its environment. In reality, organisms are restricted from niche‐tracking given biotic limitations, imperfect perception, and resource heterogeneity (Kearney & Porter, 2009). For example, in our modeling all simulated frogs survived hibernation and emergence. Yet, this may not reflect behavioral imperfections of real wood frogs, which may emerge during an early winter thaw and then succumb to later storm events (author observations, O'Connor & Rittenhouse, 2016). Furthermore, post‐emergence movement and oviposition are influenced by several additional density‐dependent processes. Early‐arriving males to a pond begin a chorus that attracts females but also additional males, increasing both the likelihood of reproduction and total fecundity (Bee, 2007). Thus, our data‐model integration here provides useful, albeit incomplete, inference into amphibian winter and spring phenology.

### Drivers of phenology across species and systems

A heightened role of exogenous factors, relative to endogenous ones, may provide optimism for our abilities to predict organismal responses to climate change. Indeed, our study suggests that interpopulation variation in phenology may arise from environmental gradients more so than from variation in biological traits. One may draw the conclusion that ubiquitous geospatial environmental datasets may thus offer useful predictors for phenology, even without scalable trait‐based information. Yet, how well might our finding of a minimal role of endogenous biological traits generalize for ecology beyond amphibians? A meta‐analysis found that 16 amphibian species had phenological responses to climate change that were two to four times stronger than trees, birds, and butterflies (Parmesan, 2007). Yet, amphibians also demonstrate mixed signals of both phenological advance and delay (Arietta et al., 2020; Parmesan, 2007), perhaps as their responses to changing thermal conditions may directionally diverge from responses to changing hydric conditions (Semlitsch, 1985; Timm et al., 2007). Thus, amphibian phenology may be more sensitive to exogenous climate than to other taxa.

Other species may exhibit stronger relationships between behavior, dispersal, and phenology in response to global change. While most amphibians are poor dispersers, larger bodied or volant (flying) animals may track resources at a large spatial scale, inducing spatial shifts instead of temporal shifts, or a combination of both (Abrahms et al., 2021). As ectotherms, amphibian body temperatures are largely regulated by environmental temperatures (Angilletta Jr., 2009). Endotherm phenology may not respond as clearly to meteorological drivers as we observed here for frogs, perhaps as responses to meteorology may be mediated through availability of food or other resources (Angilletta Jr., 2009; Porter et al., 2023; Zhang et al., 2025). Thus, we caution that our results do not easily generalize across the tree of life, given the idiosyncratic nature of species’ temporal and spatial responses to climate change (Fredston et al., 2025; Pilliod et al., 2022).

Important, therefore, is the need to represent taxonomically specific mechanistic relationships between organisms and their environment, as correlative approaches often have poor performance in an out‐of‐sample prediction such as forecasting (Briscoe et al., 2023). This includes representing environmental variables as experienced by the organisms themselves, such as near‐surface microclimates instead of weather station observations (Klinges et al., 2024). While representing the proximal conditions that organisms experience across large spatiotemporal scales remains challenging, mechanistic microclimate models serve as useful testing grounds for hypotheses on the range of thermal and hydric conditions that individuals and populations experience.

## CONCLUSION

Here, we paired long‐term field observations with trait measurements and mechanistic simulations to understand the environmental and biological processes that determine a pond‐breeding amphibian's phenological response across several decades. Combining regularly collected observations and mechanistic modeling helps build a generalizable framework by which to understand phenological responses to global change (Reinke et al., 2019). We therefore amplify recent calls to fuse field‐based observations with big data and model simulations to iteratively improve prediction and understanding for ecology and conservation (McCleery et al., 2023). Sensitivity analyses then serve as a useful avenue by which to evaluate parameter importance in a predictive framework. Given the broad modularity of the NicheMapR mechanistic models, our approach can be leveraged to explore physiological and phenological responses for diverse species and systems simultaneously (Kearney & Porter, 2017, 2020). Standardization of this approach, grounded in the mathematical representation of mechanism, builds broad understanding of how global change influences ecology for improved prediction and management.

## AUTHOR CONTRIBUTIONS

L. Kealoha Freidenburg, Adriana D. Rubinstein, and David K. Skelly collected the data. David H. Klinges and David K. Skelly conceived the study. David H. Klinges performed the analysis and wrote the manuscript. All co‐authors contributed to manuscript revisions.

## CONFLICT OF INTEREST STATEMENT

The authors declare no conflicts of interest.

## Supporting information


Appendix S1.

## Data Availability

Data and code (Klinges & Skelly, 2026) are available in Zenodo at https://doi.org/10.5281/zenodo.18281730.

## References

[ecy70394-bib-0001] Abatzoglou, J. T. 2013. “Development of Gridded Surface Meteorological Data for Ecological Applications and Modelling.” International Journal of Climatology 33: 121–131.

[ecy70394-bib-0002] Abrahms, B. , E. O. Aikens , J. B. Armstrong , W. W. Deacy , M. J. Kauffman , and J. A. Merkle . 2021. “Emerging Perspectives on Resource Tracking and Animal Movement Ecology.” Trends in Ecology & Evolution 36: 308–320. 33229137 10.1016/j.tree.2020.10.018

[ecy70394-bib-0003] Angilletta, M. J., Jr. 2009. Thermal Adaptation: A Theoretical and Empirical Synthesis. Oxford, UK: Oxford University Press.

[ecy70394-bib-0004] Arietta, A. Z. A. , L. K. Freidenburg , M. C. Urban , S. B. Rodrigues , A. Rubinstein , and D. K. Skelly . 2020. “Phenological Delay Despite Warming in Wood Frog Rana Sylvatica Reproductive Timing: A 20‐Year Study.” Ecography 43: 1791–1800.

[ecy70394-bib-0005] Baecher, J. A. , S. A. Johnson , E. A. Roznik , and B. R. Scheffers . 2023. “Experimental Evaluation of How Biological Invasions and Climate Change Interact to Alter the Vertical Assembly of an Amphibian Community.” Journal of Animal Ecology 92: 875–888. 36872563 10.1111/1365-2656.13899

[ecy70394-bib-0006] Bee, M. A. 2007. “Selective Phonotaxis by Male Wood Frogs (*Rana sylvatica*) to the Sound of a Chorus.” Behavioral Ecology and Sociobiology 61: 955–966.

[ecy70394-bib-0007] Bellis, E. D. 1965. “Home Range and Movements of the Wood Frog in a Northern Bog.” Ecology 46: 90–98.

[ecy70394-bib-0008] Benard, M. F. , and K. R. Greenwald . 2023. “Environmental Drivers of Amphibian Breeding Phenology Across Multiple Sites.” Diversity 15: 253.

[ecy70394-bib-0009] Berven, K. A. 1982. “The Genetic Basis of Altitudinal Variation in the Wood Frog *Rana sylvatica*. I. An Experimental Analysis of Life History Traits.” Evolution 36: 962–983. 28567824 10.1111/j.1558-5646.1982.tb05466.x

[ecy70394-bib-0010] Billet, L. S. , Y. A. Alshwairikh , L. K. Freidenburg , A. Rubinstein , S. Tracy , S. Nelson , and D. K. Skelly . 2024. “Long‐Term Decline of the Spotted Salamander (*Ambystoma maculatum*) in an Undeveloped Landscape.” Herpetologica 80: 11–21.

[ecy70394-bib-0011] Bolker, B. M. 2003. “Combining Endogenous and Exogenous Spatial Variability in Analytical Population Models.” Theoretical Population Biology 64: 255–270. 14522167 10.1016/s0040-5809(03)00090-x

[ecy70394-bib-0012] Briscoe, N. J. , S. D. Morris , P. D. Mathewson , L. B. Buckley , M. Jusup , O. Levy , I. M. D. Maclean , et al. 2023. “Mechanistic Forecasts of Species Responses to Climate Change: The Promise of Biophysical Ecology.” Global Change Biology 29: 1451–1470. 36515542 10.1111/gcb.16557

[ecy70394-bib-0013] Brown, R. D. , and D. A. Robinson . 2011. “Northern Hemisphere Spring Snow Cover Variability and Change Over 1922–2010 Including an Assessment of Uncertainty.” The Cryosphere 5: 219–229.

[ecy70394-bib-0014] Buckley, L. B. , E. Carrington , M. E. Dillon , C. García‐Robledo , S. B. Roberts , J. L. Wegrzyn , and M. C. Urban . 2023. “Characterizing Biological Responses to Climate Variability and Extremes to Improve Biodiversity Projections.” PLOS Climate 2: e0000226.

[ecy70394-bib-0015] Choi, G. , D. A. Robinson , and S. Kang . 2010. “Changing Northern Hemisphere Snow Seasons.” Journal of Climate 23: 5305–5310.

[ecy70394-bib-0016] Churchill, T. A. , and K. B. Storey . 1993. “Dehydration Tolerance in Wood Frogs: A New Perspective on Development of Amphibian Freeze Tolerance.” American Journal of Physiology‐Regulatory, Integrative and Comparative Physiology 265: R1324–R1332. 10.1152/ajpregu.1993.265.6.R1324 8285273

[ecy70394-bib-0017] Cleland, E. E. , I. Chuine , A. Menzel , H. A. Mooney , and M. D. Schwartz . 2007. “Shifting Plant Phenology in Response to Global Change.” Trends in Ecology & Evolution 22: 357–365. 17478009 10.1016/j.tree.2007.04.003

[ecy70394-bib-0018] CT ECO . 2016. 2016 Lidar Elevation. Storrs, CT: Capitol Region Council of Governments. http://cteco.uconn.edu/data/flight2016/index.htm.

[ecy70394-bib-0019] Dodd, C. K. 2013. Frogs of the United States and Canada, 1st ed. Baltimore, MD: Johns Hopkins University Press.

[ecy70394-bib-0020] Enriquez‐Urzelai, U. , M. R. Kearney , A. G. Nicieza , and R. Tingley . 2019. “Integrating Mechanistic and Correlative Niche Models to Unravel Range‐Limiting Processes in a Temperate Amphibian.” Global Change Biology 25: 2633–2647. 31050846 10.1111/gcb.14673

[ecy70394-bib-0021] Fitzpatrick, M. J. , W. P. Porter , J. N. Pauli , M. R. Kearney , M. Notaro , and B. Zuckerberg . 2020. “Future Winters Present a Complex Energetic Landscape of Decreased Costs and Reduced Risk for a Freeze‐Tolerant Amphibian, the Wood Frog (*Lithobates sylvaticus*).” Global Change Biology 26: 6350–6362. 32871618 10.1111/gcb.15321

[ecy70394-bib-0022] Fitzpatrick, M. J. , B. Zuckerberg , J. N. Pauli , M. R. Kearney , K. L. Thompson , L. C. Werner , and W. P. Porter . 2019. “Modeling the Distribution of Niche Space and Risk for a Freeze‐Tolerant Ectotherm, *Lithobates sylvaticus* .” Ecosphere 10: e02788.

[ecy70394-bib-0023] Fredston, A. L. , M. W. Tingley , H. C. Montague , L. J. Neate‐Clegg , L. H. Evans , N. C. Antão , I.‐C. C. Ban , et al. 2025. “Reimagining Species on the Move Across Space and Time.” Trends in Ecology & Evolution 40: 629–638. 40345938 10.1016/j.tree.2025.03.015

[ecy70394-bib-0024] Gareth, J. , W. Daniela , H. Trevor , T. Robert , and T. Jonathan . 2013. An Introduction to Statistical Learning, 1st ed. New York: Springer.

[ecy70394-bib-0025] Groff, L. A. , A. J. K. Calhoun , and C. S. Loftin . 2016. “Hibernal Habitat Selection by Wood Frogs (*Lithobates sylvaticus*) in a Northern New England Montane Landscape.” Journal of Herpetology 50: 559–569.

[ecy70394-bib-0026] Groff, L. A. , A. J. K. Calhoun , and C. S. Loftin . 2017. “Amphibian Terrestrial Habitat Selection and Movement Patterns Vary with Annual Life‐History Period.” Canadian Journal of Zoology 95: 433–442.

[ecy70394-bib-0027] Holmquist, J. R. , L. Windham‐Myers , B. Bernal , K. B. Byrd , S. Crooks , M. E. Gonneea , N. Herold , et al. 2018. “Uncertainty in United States Coastal Wetland Greenhouse Gas Inventorying.” Environmental Research Letters 13: 115005.

[ecy70394-bib-0028] Homer, C. G. , J. A. Fry , and C. A. Barnes . 2012. The National Land Cover Database. Page Fact Sheet. Reston, VA: U.S. Geological Survey. https://pubs.usgs.gov/publication/fs20123020.

[ecy70394-bib-0029] Huey, R. B. , M. R. Kearney , A. Krockenberger , J. A. M. Holtum , M. Jess , and S. E. Williams . 2012. “Predicting Organismal Vulnerability to Climate Warming: Roles of Behaviour, Physiology and Adaptation.” Philosophical Transactions of the Royal Society B: Biological Sciences 367: 1665–1679. 10.1098/rstb.2012.0005 PMC3350654 22566674

[ecy70394-bib-0030] Kearney, M. , and W. Porter . 2009. “Mechanistic Niche Modelling: Combining Physiological and Spatial Data to Predict Species' Ranges.” Ecology Letters 12: 334–350. 19292794 10.1111/j.1461-0248.2008.01277.x

[ecy70394-bib-0031] Kearney, M. R. , and W. P. Porter . 2017. “NicheMapR—An R Package for Biophysical Modelling: The Microclimate Model.” Ecography 40: 664–674.

[ecy70394-bib-0032] Kearney, M. R. , and W. P. Porter . 2020. “NicheMapR—An R Package for Biophysical Modelling: The Ectotherm and Dynamic Energy Budget Models.” Ecography 43: 85–96.

[ecy70394-bib-0033] Klinges, D. , and D. Skelly . 2026. “Data and Code for: Meteorology and Geography, More Than Biological Traits, Drive Variation in Frog Phenology Across Decades.” Zenodo. 10.5281/zenodo.18281730 42087364

[ecy70394-bib-0034] Klinges, D. H. , J. A. Baecher , J. J. Lembrechts , I. M. D. Maclean , J. Lenoir , C. Greiser , M. Ashcroft , et al. 2024. “Proximal Microclimate: Moving Beyond Spatiotemporal Resolution Improves Ecological Predictions.” Global Ecology and Biogeography 33: e13884.

[ecy70394-bib-0035] Lang, W. , Y. Zhang , X. Li , F. Meng , Q. Liu , K. Wang , H. Xu , et al. 2024. “Phenological Divergence Between Plants and Animals Under Climate Change.” Nature Ecology & Evolution 9: 261–272. 39653762 10.1038/s41559-024-02597-0

[ecy70394-bib-0036] Lee‐Yaw, J. A. , J. T. Irwin , and D. M. Green . 2008. “Postglacial Range Expansion from Northern Refugia by the Wood Frog, Rana Sylvatica.” Molecular Ecology 17: 867–884. 18179428 10.1111/j.1365-294X.2007.03611.x

[ecy70394-bib-0037] Loughnan, D. , S. Joly , G. Legault , H. M. Kharouba , M. Betancourt , and E. M. Wolkovich . 2024. “Phenology Varies with Phylogeny but Not by Trophic Level with Climate Change.” Nature Ecology & Evolution 8: 1889–1896. 39232116 10.1038/s41559-024-02499-1

[ecy70394-bib-0038] Ma, L. , S. R. Conradie , C. L. Crawford , A. S. Gardner , M. R. Kearney , I. M. D. Maclean , A. E. McKechnie , C. R. Mi , R. A. Senior , and D. S. Wilcove . 2023. “Global Patterns of Climate Change Impacts on Desert Bird Communities.” Nature Communications 14: 211. 10.1038/s41467-023-35814-8 PMC9839677 36639376

[ecy70394-bib-0039] Maclean, I. M. D. , and D. H. Klinges . 2021. “Microclimc: A Mechanistic Model of Above, Below and Within‐Canopy Microclimate.” Ecological Modelling 451: 109567.

[ecy70394-bib-0040] Malmborg, C. A. , A. M. Willson , L. M. Bradley , M. A. Beatty , D. H. Klinges , G. Koren , A. S. L. Lewis , K. Oshinubi , and W. M. Woelmer . 2024. “Defining Model Complexity: An Ecological Perspective.” Meteorological Applications 31: e2202.

[ecy70394-bib-0041] McCleery, R. , R. Guralnick , M. Beatty , M. Belitz , C. J. Campbell , J. Idec , M. Jones , Y. Kang , A. Potash , and R. J. Fletcher, Jr. 2023. “Uniting Experiments and Big Data to Advance Ecology and Conservation.” Trends in Ecology & Evolution 38: 970–979. 37330409 10.1016/j.tree.2023.05.010

[ecy70394-bib-0042] Muñoz, M. M. , G. M. Langham , M. C. Brandley , D. F. Rosauer , S. E. Williams , and C. Moritz . 2016. “Basking Behavior Predicts the Evolution of Heat Tolerance in Australian Rainforest Lizards.” Evolution 70: 2537–2549. 27612295 10.1111/evo.13064

[ecy70394-bib-0043] Neate‐Clegg, M. H. C. , B. A. Tonelli , and M. W. Tingley . 2024. “Advances in Breeding Phenology Outpace Latitudinal and Elevational Shifts for North American Birds Tracking Temperature.” Nature Ecology & Evolution 8: 2027–2036. 39223395 10.1038/s41559-024-02536-z

[ecy70394-bib-0044] O'Connor, J. H. , and T. A. G. Rittenhouse . 2016. “Snow Cover and Late Fall Movement Influence Wood Frog Survival During an Unusually Cold Winter.” Oecologia 181: 635–644. 26497126 10.1007/s00442-015-3450-z

[ecy70394-bib-0045] Palmer, W. C. 1965. Meteorological Drought. Office of Climatology Research Paper No. 45 U.S. Washington, DC: Department of Commerce, Weather Bureau.

[ecy70394-bib-0046] Parmesan, C. 2006. “Ecological and Evolutionary Responses to Recent Climate Change.” Annual Review of Ecology, Evolution, and Systematics 37: 637–669.

[ecy70394-bib-0047] Parmesan, C. 2007. “Influences of Species, Latitudes and Methodologies on Estimates of Phenological Response to Global Warming.” Global Change Biology 13: 1860–1872.

[ecy70394-bib-0048] Pauli, J. N. , B. Zuckerberg , J. P. Whiteman , and W. Porter . 2013. “The Subnivium: A Deteriorating Seasonal Refugium.” Frontiers in Ecology and the Environment 11: 260–267.

[ecy70394-bib-0049] Piao, S. , Q. Liu , A. Chen , I. A. Janssens , Y. Fu , J. Dai , L. Liu , X. Lian , M. Shen , and X. Zhu . 2019. “Plant Phenology and Global Climate Change: Current Progresses and Challenges.” Global Change Biology 25: 1922–1940. 30884039 10.1111/gcb.14619

[ecy70394-bib-0050] Pilliod, D. S. , R. M. McCaffery , R. S. Arkle , R. D. Scherer , J. B. Cupples , L. A. Eby , B. R. Hossack , et al. 2022. “Importance of Local Weather and Environmental Gradients on Demography of a Broadly Distributed Temperate Frog.” Ecological Indicators 136: 108648.

[ecy70394-bib-0051] Porter, W. P. , A. E. Bertz , P. D. Mathewson , L. C. Solorzano , P. N. Dudley , R. Bonazza , and K. G. Gebremedhin . 2023. “Climate Spaces and Cliffs: A Novel Bovine Thermodynamic and Mass Balances Model.” Animals 13: 3043. 37835649 10.3390/ani13193043 PMC10572002

[ecy70394-bib-0052] Primack, R. B. , A. S. Gallinat , E. R. Ellwood , T. M. Crimmins , M. D. Schwartz , M. D. Staudinger , and A. J. Miller‐Rushing . 2023. “Ten Best Practices for Effective Phenological Research.” International Journal of Biometeorology 67: 1509–1522. 37507579 10.1007/s00484-023-02502-7 PMC10457241

[ecy70394-bib-0053] R Core Team . 2024. A Language and Environment for Statistical Computing. Vienna, Austria: R Foundation for Statistical Computing.

[ecy70394-bib-0054] Randin, C. F. , M. B. Ashcroft , J. Bolliger , J. Cavender‐Bares , N. C. Coops , S. Dullinger , T. Dirnböck , et al. 2020. “Monitoring Biodiversity in the Anthropocene Using Remote Sensing in Species Distribution Models.” Remote Sensing of Environment 239: 111626.

[ecy70394-bib-0055] Reinke, B. A. , D. A. W. Miller , and F. J. Janzen . 2019. “What Have Long‐Term Field Studies Taught us About Population Dynamics?” Annual Review of Ecology, Evolution, and Systematics 50: 261–278.

[ecy70394-bib-0056] Relyea, R. A. 2002. “Local Population Differences in Phenotypic Plasticity: Predator‐Induced Changes in Wood Frog Tadpoles.” Ecological Monographs 72: 77–93.

[ecy70394-bib-0057] Rowland, F. E. , E. S. Schyling , L. K. Freidenburg , M. C. Urban , J. L. Richardson , A. Z. A. Arietta , S. B. Rodrigues , A. D. Rubinstein , M. F. Benard , and D. K. Skelly . 2022. “Asynchrony, Density Dependence, and Persistence in an Amphibian.” Ecology 103: e3696. 35352342 10.1002/ecy.3696

[ecy70394-bib-0058] Semlitsch, R. D. 1985. “Analysis of Climatic Factors Influencing Migrations of the Salamander *Ambystoma talpoideum* .” Copeia 1985: 477–489.

[ecy70394-bib-0059] Shekhovtsov, S. V. , N. A. Bulakhova , Y. P. Tsentalovich , E. A. Zelentsova , L. V. Yanshole , E. N. Meshcheryakova , and D. I. Berman . 2020. “Metabolic Response of the Siberian Wood Frog *Rana amurensis* to Extreme Hypoxia.” Scientific Reports 10: 14604. 32884088 10.1038/s41598-020-71616-4 PMC7471963

[ecy70394-bib-0060] Sinclair, B. J. , J. R. Stinziano , C. M. Williams , H. A. MacMillan , K. E. Marshall , and K. B. Storey . 2013. “Real‐Time Measurement of Metabolic Rate During Freezing and Thawing of the Wood Frog, *Rana sylvatica*: Implications for Overwinter Energy Use.” Journal of Experimental Biology 216: 292–302. 23255194 10.1242/jeb.076331

[ecy70394-bib-0061] Skelly, D. K. , L. K. Freidenburg , and J. M. Kiesecker . 2002. “Forest Canopy and the Performance of Larval Amphibians.” Ecology 83: 983–992.

[ecy70394-bib-0062] Skelly, D. K. , E. E. Werner , and S. A. Cortwright . 1999. “Long‐Term Distributional Dynamics of a Michigan Amphibian Assemblage.” Ecology 80: 2326–2337.

[ecy70394-bib-0063] Slatyer, R. A. , K. D. L. Umbers , and P. A. Arnold . 2022. “Ecological Responses to Variation in Seasonal Snow Cover.” Conservation Biology 36: e13727. 33636757 10.1111/cobi.13727

[ecy70394-bib-0064] Stoler, A. B. , J. P. Stephens , R. A. Relyea , K. A. Berven , and S. D. Tiegs . 2015. “Leaf Litter Resource Quality Induces Morphological Changes in Wood Frog (*Lithobates sylvaticus*) Metamorphs.” Oecologia 179: 667–677. 26188520 10.1007/s00442-015-3387-2

[ecy70394-bib-0065] Storey, K. B. , and J. M. Storey . 1984. “Biochemical Adaption for Freezing Tolerance in the Wood Frog, *Rana sylvatica* .” Journal of Comparative Physiology B 155: 29–36.

[ecy70394-bib-0066] Sunday, J. M. , A. E. Bates , M. R. Kearney , R. K. Colwell , N. K. Dulvy , J. T. Longino , and R. B. Huey . 2014. “Thermal‐Safety Margins and the Necessity of Thermoregulatory Behavior Across Latitude and Elevation.” Proceedings of the National Academy of Sciences 111: 5610–5615. 10.1073/pnas.1316145111 PMC3992687 24616528

[ecy70394-bib-0067] Thornton, P. E. , R. Shrestha , M. Thornton , S.‐C. Kao , Y. Wei , and B. E. Wilson . 2021. “Gridded Daily Weather Data for North America with Comprehensive Uncertainty Quantification.” Scientific Data 8: 190. 34301954 10.1038/s41597-021-00973-0 PMC8302764

[ecy70394-bib-0068] Timm, B. C. , K. McGarigal , and B. W. Compton . 2007. “Timing of Large Movement Events of Pond‐Breeding Amphibians in Western Massachusetts, USA.” Biological Conservation 136: 442–454.

[ecy70394-bib-0069] Urban, M. C. , P. L. Zarnetske , and D. K. Skelly . 2013. “Moving Forward: Dispersal and Species Interactions Determine Biotic Responses to Climate Change.” Annals of the New York Academy of Sciences 1297: 44–60. 23819864 10.1111/nyas.12184

[ecy70394-bib-0070] Wainwright, P. C. , and S. M. Reilly . 1994. Ecological Morphology: Integrative Organismal Biology. Chicago, IL: University of Chicago Press.

[ecy70394-bib-0071] Willems, F. M. , J. F. Scheepens , C. Ammer , S. Block , A. Bucharova , P. Schall , M. Sehrt , and O. Bossdorf . 2021. “Spring Understory Herbs Flower Later in Intensively Managed Forests.” Ecological Applications 31: e02332. 33765327 10.1002/eap.2332

[ecy70394-bib-0072] Wipf, S. , and C. Rixen . 2010. “A Review of Snow Manipulation Experiments in Arctic and Alpine Tundra Ecosystems.” Polar Research 29: 95–109.

[ecy70394-bib-0073] Zhang, Y. , P. D. Mathewson , W. P. Porter , and Q. Zhang . 2025. “Mechanistically Simulating the Effects of Climate Change to Identify Conservation Hotspots and Reproduction Potential for an Endangered Species.” Biological Conservation 302: 110905.

[ecy70394-bib-0074] Zuckerberg, B. , and J. N. Pauli . 2018. “Conserving and Managing the Subnivium.” Conservation Biology 32: 774–781. 29420843 10.1111/cobi.13091

