# Supplementary material for: Meteorology and geography, more than biological traits, drive variation in frog phenology across decades
Source: Ecology. 2026 May 5;107:e70394. doi: 10.1002/ecy.70394 (PMC13144712; doi:10.1002/ecy.70394)
Supplement: Supplementary file 1 — Appendix S1. [file ECY-107-e70394-s001.pdf]

Appendix S1

Meteorology and geography, more than biological traits, drive variation in frog phenology across decades

David H. Klinges, L. Kealoha Freidenburg, Adriana D. Rubinstein, David K. Skelly

Ecology

Software

All analyses were conducted in R v4.2 (R Core Team 2024). The following R packages were employed (yet not including the many important dependencies for each of these packages): the *tidyverse* suite of packages (in particular *dplyr, ggplot2,* and *lubridate*) for importing, curating, and visualizing data (Wickham 2017); *terra* (Hijmans et al. 2022) and *zoo* (Zeileis et al. 2022) for spatiotemporal data processing; *mcera5* (Klinges et al., 2022) for accessing ERA5 climate data from the ECMWF portal; *NicheMapR* (Kearney and Porter 2017, 2020) for microclimate and biophysical modeling; *elevatR* (Hollister et al. 2022) and *microclima* (Maclean et al. 2019) for efficient access of digital elevation models; *doParallel* (Daniel et al. 2022) for threaded processing; *lme4* (Bates et al. 2015) for fitting linear mixed-effects models; *randomForest* for fitting random forest model (Liaw and Wiener 2002); *gridExtra* (Auguie and Antonov 2017) and *RColorBrewer* (Neuwirth 2022) for visualization; and *conflicted* (Wickham and RStudio 2021) for function name conflict handling. We thank the efforts of many for producing open-source software to advance research efforts.

Supporting Figures

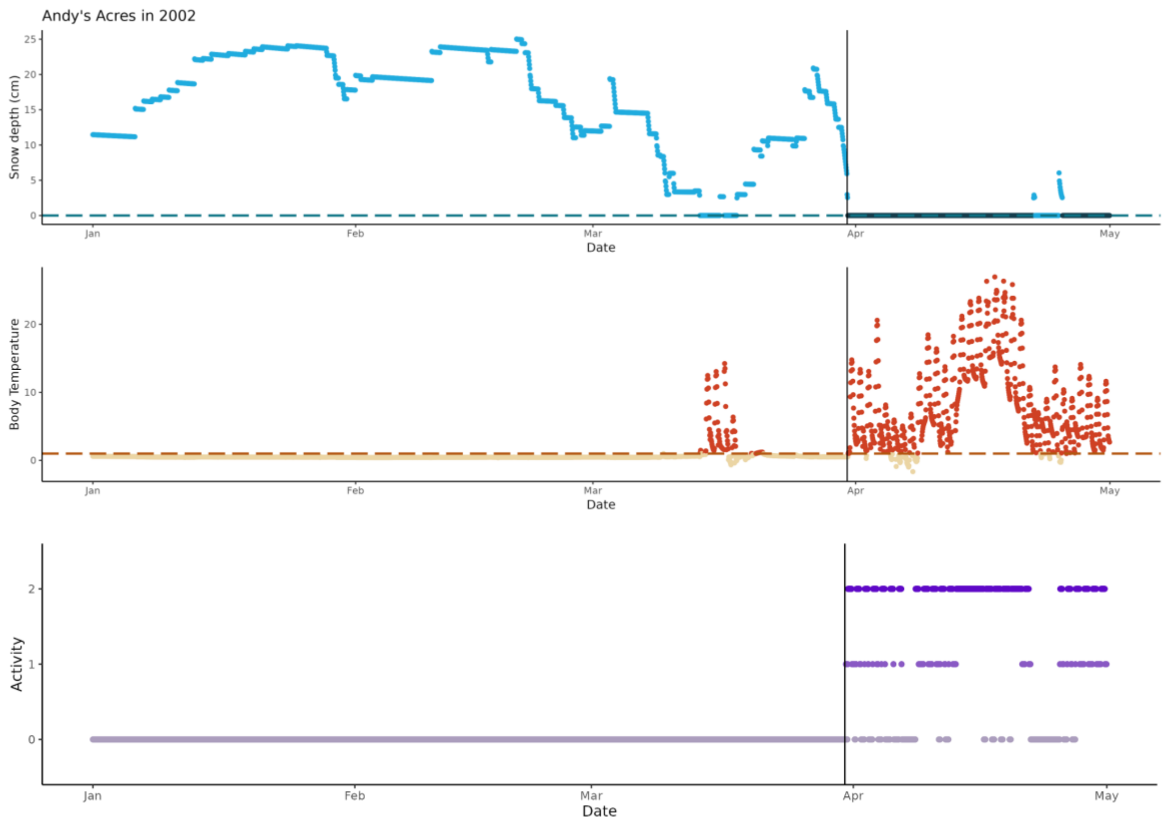

Figure S1. Example time series of snow depth, frog body temperatures, and frog activity levels from the pond Andy's Acres in 2002, to demonstrate rules used in simulations to determine frog emergence date (black lines). Only once both modeled snow depths dropped below a given threshold (0cm) for a set amount of time (7 days), and modeled body temperatures increased above a given threshold (-0.16 °C) for 12 consecutive hours, was frog activity allowed to be induced (0 = inactive, 1 = basking, 2 = foraging), at which point emergence was designated.

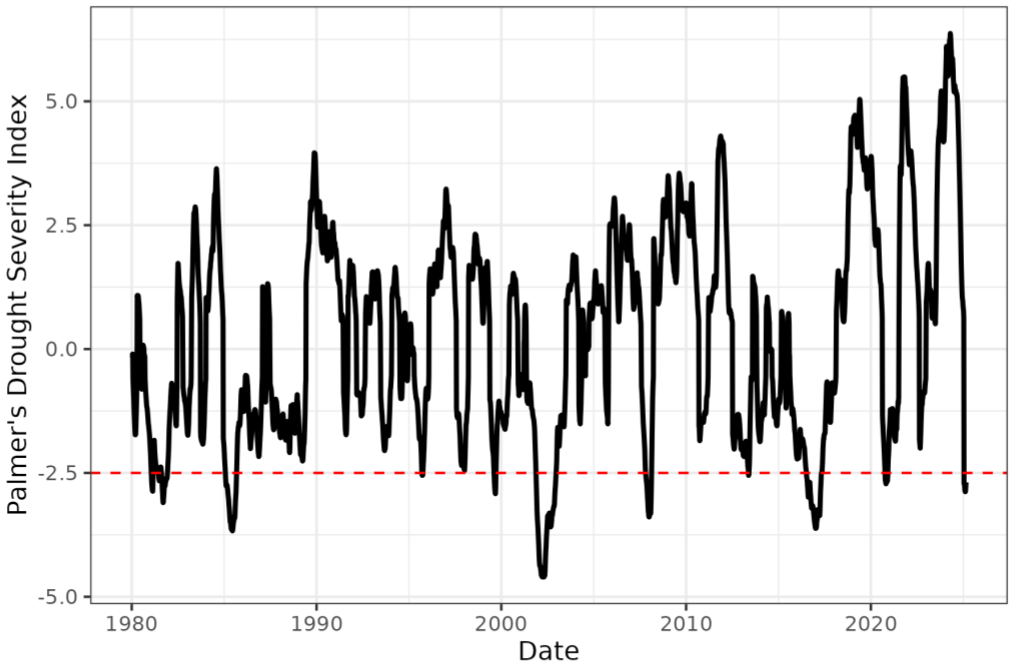

Figure S2. Palmer's Drought Severity Index, as estimated by GridMet meteorology, for Yale Myers Forest in Connecticut, USA.

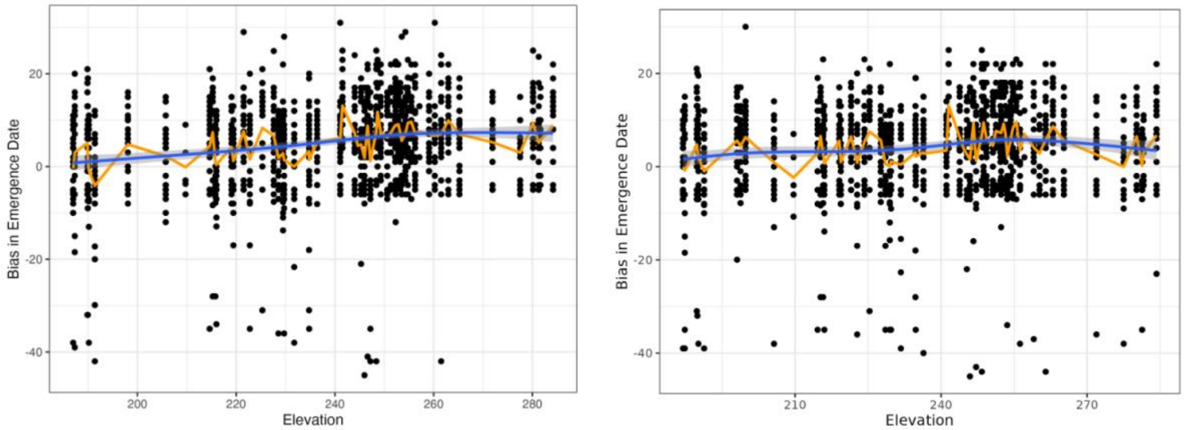

Figure S3. Left: use of average elevation for each pond in frog emergence modeling introduced a bias of delayed emergence with increased elevation. Right: Setting all ponds to the site-wide average of elevation (236 m ASL) reduced this bias.

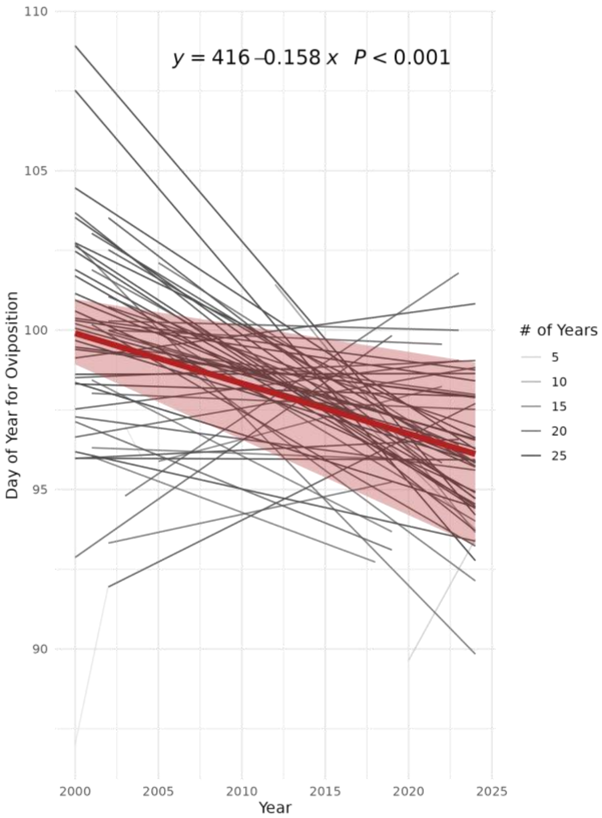

Figure S4. Linear mixed-effects model predictions of the per-pond (grey lines) and average (red line; ribbon represents 95% confidence intervals) trend in emergence day across 25 years. Shading represents the number of years of observations per pond.

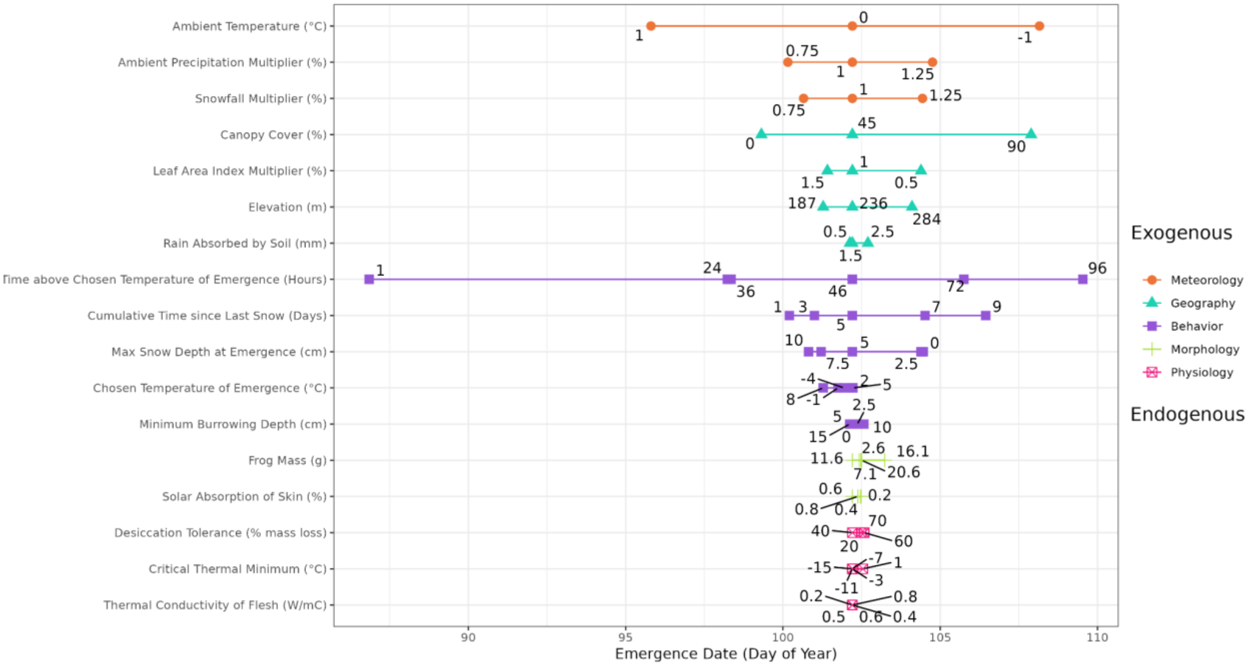

Figure S5. Sensitivity of adult frog post-hibernation emergence to a range of exogenous and endogenous parameters, with NicheMapR's dynamic energy budget model turned on. Each point indicates the average emergence day-of-year for a single frog across a 45-year simulation (1980 - 2024). Quantitative labels on each colored segment indicate the value of the input parameter for that simulation; all other parameters were held at their average value (i.e. label of middle point for each segment). Increasing width of a segment entails stronger importance of a parameter for driving frog emergence. Exogenous parameters (classified as meteorology or geography) generally were more important than endogenous parameters (physiology, morphology, behavior) for driving variation in emergence day. See Table S3 for an explanation of all parameters.

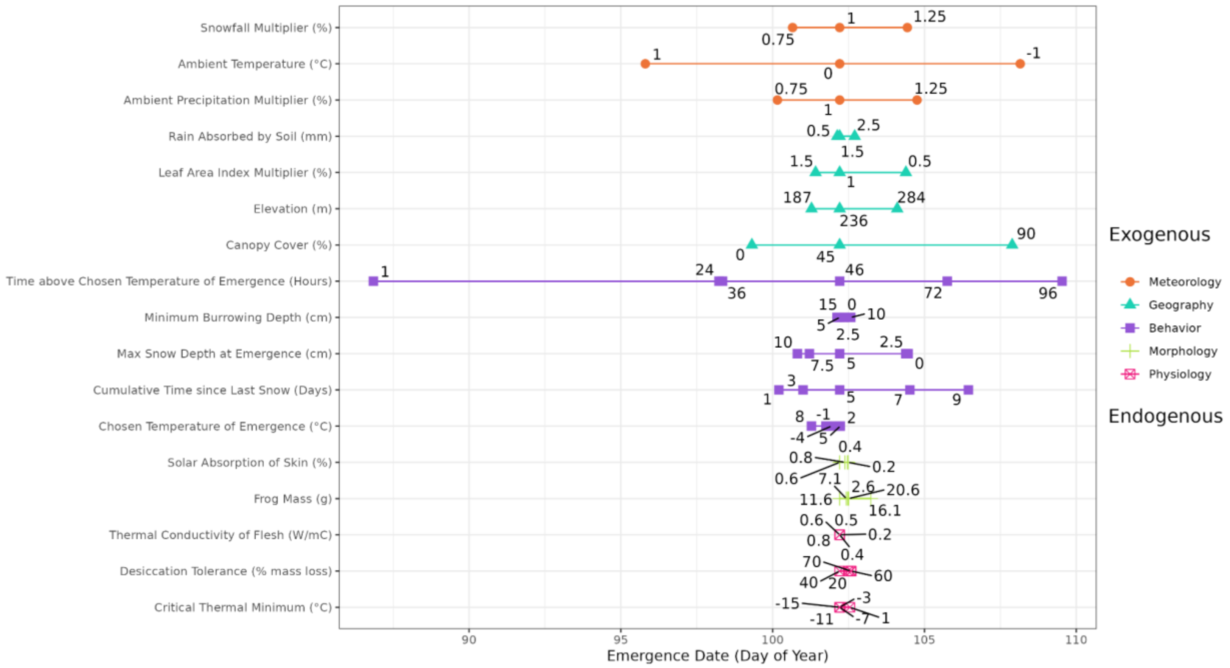

Figure S6. Sensitivity analysis of frog emergence dates, but with inflated ranges of biological trait values. Each point indicates the average emergence day-of-year for a single frog across a 45-year simulation (1980 - 2024). Quantitative labels on each colored segment indicate the value of the input parameter for that simulation; all other parameters were held at their average value (i.e. label of middle point for each segment). Increasing width of a segment entails stronger importance of a parameter for driving frog emergence. Exogenous parameters (classified as meteorology or geography) generally were more important than endogenous parameters (physiology, morphology, behavior) for driving variation in emergence day, with the exception of the behavioral parameter of the number of hours above the chosen temperature of emergence, which explained 31.7% of all variation. See Table S3 for an explanation of all parameters.

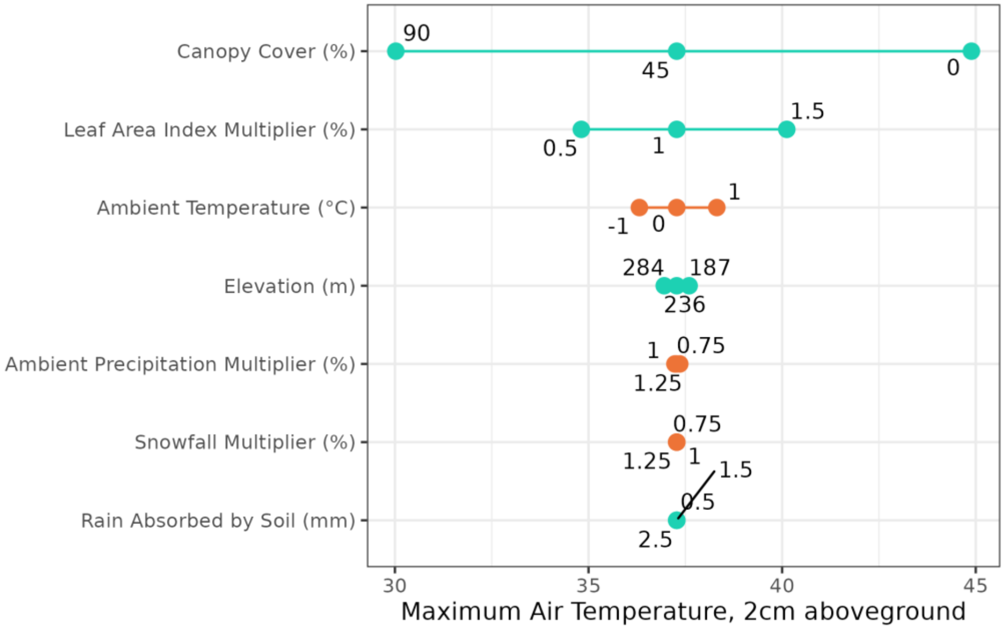

Figure S7. Sensitivity of maximum soil temperature (2 cm aboveground) to meteorological and geographical parameters. Each point indicates the average minimum soil temperature across a 45-year simulation (1980 - 2024). Quantitative labels on each colored segment indicate the value of the input parameter for that simulation; all other parameters were held at their average value (i.e. label of middle point for each segment).

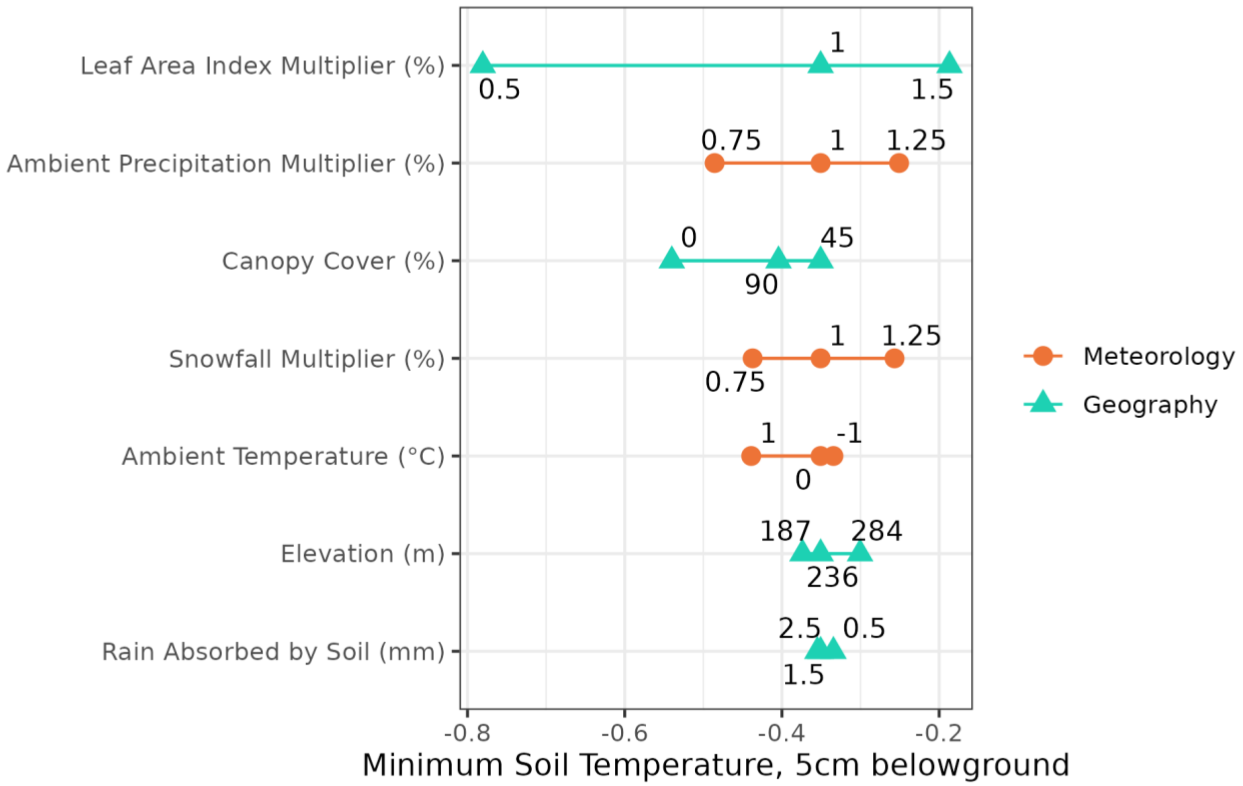

Figure S8. Sensitivity of minimum soil temperature (5 cm belowground) to meteorological and geographical parameters. Each point indicates the average minimum soil temperature across a 45-year simulation (1980 - 2024). Quantitative labels on each colored segment indicate the value of the input parameter for that simulation; all other parameters were held at their average value (i.e. label of middle point for each segment).

Supporting Tables

Table S1. Inputs for the NicheMapR mechanistic microclimate model and their sources.

Input Name	Description	Source
Meteorology	gridMET estimates of daily surface meteorology at ~4-km spatial resolution	Abatzoglou 2013
Digital Elevation model	Lidar-derived digital elevation model from flights in 2016 0.8-m digital elevation model of Connecticut, for estimates of elevation, slope, aspect, and topographic wetness index	Connecticut Environmental Conditions Online (CT ECO) 2016
Landscape-level canopy cover / shading level	Percent forest cover estimates within a 200-m radius around each pond, derived from 30-m resolution imagery from 2019. We set minimum shade as 5% less than the percent forest cover surrounding a pond, and maximum shade as 5% greater than per-pond percent forest cover	Billet et al. 2024, US Geological Survey National Land Cover Database (Homer et al. 2012)
Pond-specific canopy cover / shading level	Empirical estimates of minimum and maximum canopy cover from hemispherical photographs taken during both leaf-on and leaf-off conditions. Measured in 1999, 2012, and 2017.	Arietta et al. 2020, Halverson et al. 2003
Vegetation Properties	Leaf area index, leaf orientation relative to a vertical plane, canopy height, roughness height, heat transfer roughness height, zero plane displacement correction factor; all values as estimated for mixed forest	Maclean and Klinges 2021
Soil properties	Soil bulk density (Mg/m3) and proportions of sand, silt, and clay at depths from 0 cm to 200 cm; hydraulic properties estimated from physical properties	Campbell 1985, Enriquez-Urzelai et al. 2019, Cheng et al. 2022

Table S2. Inputs for the mechanistic ectotherm model used for validation, and their sources. All inputs not listed here were left as defaults.

Input Type	NicheMapR ectotherm() argument name	Description	Value	Source / Justification
Physiology	T_pref	Thermal preference, °C	26.0	Fitzpatrick et al. 2019, co-author field observations of wood frogs
Physiology	T_RB_min	Minimum body temperature at which animal will move from retreat to seek a basking site, °C	2.0	Fitzpatrick et al. 2019, co-author field observations of wood frogs
Physiology	T_F_max	Maximum body temperature at which foraging occurs, °C	30.0	Fitzpatrick et al. 2019, co-author field observations of wood frogs
Physiology	CT_max	Critical thermal maximum, °C	39.0	Skelly and Freidenburg 2000
Physiology	CT_min	Critical thermal minimum, °C	-8.0	Costanzo et al. 2013
Physiology	CT_minthresh	Number of consecutive hours below CT_min that causes death	20	Costanzo et al. 2013
Physiology	CT_kill	Animal dies when it hits thermal limits? 1 = yes, 0 = no	1	Realistic value for wood frogs
Physiology	pct_H_R	Maximum tolerated dehydration for foraging (prohibits foraging if greater than this, %)	25	Realistic value for aquatic anuran, e.g Tracy et al. 2010
Physiology	pct_H_death	Maximum tolerated dehydration (causes death if greater than this, %)	50	Realistic value for aquatic anuran, e.g. Tracy et al. 2010
Physiology	raindrink	Rainfall level (mm) at which rehydration from drinking occurs	2	Realistic value for wood frogs
Physiology	F_O2	Oxygen extraction efficiency, for respiratory water loss, %	8	Fitzpatrick et al. 2019
Physiology	K_skin	Hydraulic conducting of skin – drives liquid water exchange with substrate, kg / (m2 * s * (J / kg))	2.8×10^{-9}	Kearney et al. 2020
Physiology	kap_X	Digestive efficiency, 0 - 1	0.85	Fitzpatrick et al. 2019
Physiology	pct_H_P	Water in faeces, %	25	Fitzpatrick et al. 2019

Physiology	pct_cond	Percentage of animal surface contacting the substrate, %	7	Fitzpatrick et al. 2019
Physiology	gutfill	Percent of gut fill at which satiation occurs, %	75	Realistic value for wood frogs
Physiology	V_init	Initial structure volume, cm^3	1.90255	Kearney et al. 2020
Physiology	E_init	Initial reserve density, J / cm^3	99288.04	Kearney et al. 2020
Physiology	E_H_init	Initial maturity, J	502302	Kearney et al. 2020
Physiology	T_B_min	Minimum basking temperature, °C	2	Fitzpatrick et al. 2019, co-author field observations of wood frogs
Physiology	T_F_min	Minimum foraging temperature, °C	7	Fitzpatrick et al. 2019, co-author field observations of wood frogs
Behavior	mindepth	Minimum depth allowed when burrowing, cm	2.5	Sinclair et al. 2013, O'Connor and Rittenhouse 2016, Groff et al. 2016
Behavior	maxdepth	Maximum depth allowed when burrowing, cm	10	Sinclair et al. 2013, O'Connor and Rittenhouse 2016, Groff et al. 2016
Behavior	shade_seek	Does the organism have the ability to seek shade? 1 = yes, 0 = no	1	Realistic value for wood frogs
Behavior	burrow	Can the organism burrow? 1 = yes, 0 = no	1	Realistic value for wood frogs
Behavior	shdburrow	Choose if the animal's retreat is in the open (0), in the shade when above or below CTmin in sun (1) or in shade always (2)	1	Realistic value for wood frogs
Behavior	climb	Can the organism climb to thermoregulate? 1 = yes, 0 = no	0	Realistic value for wood frogs
Behavior	nocturn	Does the organism have nocturnal activity? 1 = yes, 0 = no	1	Realistic value for wood frogs, based on co-author observations
Behavior	crepus	Does the organism have crepuscular activity? 1 = yes, 0 = no	1	Realistic value for wood frogs, based on co-author observations
Behavior	diurn	Does the organism have diurnal activity? 1 = yes, 0 = no	1	Realistic value for wood frogs, based on co-author observations
Behavior	aquabask	If aquatic, does the organism bask? 0 = no, stay at water temp, 1 = yes, when not hungry, 2 = all the time	1	Realistic value for wood frogs

Morphology	Ww_g	Adult mass, g	11.573	Fitzpatrick et al. 2019
Morphology	shape	Organism shape. 4 = frog (measurements from a leopard frog)	4	Built-in option for frogs
Morphology	alpha_min	Minimum solar absorptivity, %	0.86	Fitzpatrick et al. 2019
Morphology	alpha_max	Maximum solar absorptivity, %	0.86	Fitzpatrick et al. 2019
Morphology	K_flesh	Thermal conductivity of flesh, W/mC	0.5	Fitzpatrick et al. 2019
Morphology	c_body	Specific heat of flesh, J / (kg - K)	4185	Fitzpatrick et al. 2019
Morphology	rho_body	Density of flesh, kg/m3	877.06	Fitzpatrick et al. 2019
Morphology	pct_wet	Percent of surface area acting as a free-water exchanger, %	100	Realistic value for wood frogs

Table S3. Variables modified for sensitivity analysis, along with the range of values used as input for models. The column 'Notes' provides justification and context for the choice of variable and the range of values.

Category	Factor Name	Description	Min. Value	Mean Value	Max. Value	Notes
Meteorology	Warming (°C)	Constant added to all hourly macroclimate temperatures	-1	0	1	While this range of temperatures is intuitive, it also approximately reflects the range of mean annual temperature (2.4 °C) derived from ERA5 (1998 - 2023) for the study site
Meteorology	Total Precipitation Multiplier (%)	Factor by which input precipitation from GridMet is multiplied to simulate more or less total precipitation. Includes snow and rain	.75	1	1.25	Interannual range of mean annual precipitation, derived from ERA5 (1998 - 2023) for the study site
Meteorology	Snowfall Multiplier (%)	Factor by which NicheMapR-modeled snowfall is multiplied to simulate more or less snow	.75	1	1.25	Same range of conditions as Total Precipitation Multiplier
Geography	Elevation (m ASL)	Elevation for both hibernacula and pond	187	236	284	Range of elevations for ponds studied in Yale Myers Forest, CT
Geography	Minimum canopy cover	The minimum canopy cover available to the animal for thermoregulation	0	45	90	Minimum and maximum canopy cover were varied in tandem, to measure their collective effect rather than isolated roles
Geography	Maximum canopy cover	The maximum canopy cover available to the animal for thermoregulation	10	55	100	
Geography	Leaf Area Index Multiplier	Leaf Area Index (LAI) is a dimensionless quantity representing the total green leaf area per unit ground surface area.	0.5	1	1.5	LAI and wind speed factor were varied in tandem, to measure their collective effect (simulating denser forest) rather

		LAI was allowed to vary seasonally 1.27 and 1.96 corresponding to mixed forest (Maclean and Klinges 2021); this parameter served as a multiplier applied to seasonally-variable LAI				than isolated roles
Geography	Wind Speed Factor	A factor by which wind speeds are multiplied, so as to simulate forest	0.3	0.75	1	
Geography	Percent Rain Absorbed by Soil (mm)	mm of rainfall causing the ground to be 90% wet for the day	0.5	1.5	2.5	Estimated range to represent well-drained versus poorly-drained soils
Physiology	CTmin (°C)	Critical thermal minimum: minimum temperature that the frog can survive for 20 hours	-11	-8	-5	Realistic range of possible CTmin values as drawn from prior studies across the spatial distribution of wood frogs (Larson et al. 2014, O'Connor and Rittenhouse 2016)
Physiology	Thermal Conductance of Flesh (W / m °C)	Rate of heat transfer from the environment to the frog, typically varies for ectotherms according to tissue composition	0.4	0.5	0.6	Realistic range of possible values for the flesh of ectotherms (Kearney et al. 2020)
Physiology	Desiccation Tolerance for Foraging	Percent of body mass lost at which point the animal stops foraging	6.7	23.3	40	Realistic range of tolerable desiccation for adult wood frogs during hibernation (Churchill and Storey 1993). Foraging- and survival-related desiccation tolerance thresholds were varied in tandem
Physiology	Desiccation Tolerance for Survival	Percent of body mass lost at which point the animal dies	20	40	60	
Morphology	Mass (g)	Adult frog mass	7.573	11.573	15.573	Realistic range of wood frog masses for northeastern populations of wood frogs (Martof and Humphries 1959,

						Werner and McCune 1979)
Morphology	Solar Absorption of Skin (%)	Percent of absorbed solar radiation by the frog's skin, which varies with skin color	60	75	90	Realistic range of possible values for skin absorption for ectothermic animals (Kearney et al. 2020, Meyer et al. 2023 Ecosphere)
Behavior	Chosen Temperature of Emergence (°C)	Coldest body temperature at which frog emerges from hibernaculum	-1	2	5	Realistic estimated range from empirical studies of wood frog freezing and reanimation (Sinclair et al. 2013, Groff et al. 2016, O'Connor and Rittenhouse 2016)
Behavior	Time above Chosen Temperature of Emergence	Cumulative hours above the threshold for chosen temperature, at which point emergence occurs	1	12	24	Estimated range
Behavior	Tolerable maximum snow depth for emergence (cm)	Maximum tolerable snow depth during emergence	0	2.5	5	Realistic estimated range from empirical studies of wood frog freezing and reanimation (Sinclair et al. 2013, Groff et al. 2016, O'Connor and Rittenhouse 2016)
Behavior	Time above tolerable maximum snow depth for emergence	Cumulative days above the threshold for maximum snow depth, at which point emergence occurs	3	7.5	12	Estimated range
Behavior	Minimum Burrowing Depth for Hibernacula	Deepest that the frog can burrow (cm)	1	2.5	5	Minimum and maximum burrowing depths were varied in tandem. Reflects realistic range of hibernacula depths for wood frogs (Groff et al. 2016)
Behavior	Maximum Burrowing Depth for Hibernacula	Deepest that the frog can burrow (cm)	10	15	20	

Table S4. Validation results for GridMet precipitation (mm), using weather station data observed in Yale Myers Forest.

**Resolution**	**Year**	**Precip RMSE**	**Precip MAE**
Daily	2017	6.85	2.84
Daily	2018	9.24	3.97
Daily	2019	5.9	2.51
Daily	2020	4.58	1.91
Daily	All	7.21	2.97

Table S5. Validation results for NicheMapR snow fall and snow depth (cm), using data observed from a NOAA Global Historical Climatology Network weather station in Eastford, CT.

Resolution	Year	Snowfall RMSE	Snowfall MAE	Snow Depth RMSE	Snow Depth MAE
Daily	2016	1.55	1.06	4.13	2.51
Daily	2017	10.37	4.95	14.29	14.11
Daily	2018	7.76	4.68	0.6	0.6
Daily	2019	9.59	5.7	23.22	19.89
Daily	2020	8.01	5.35	10.38	7.97
Daily	2021	2.4	0.43	11.87	9.86
Daily	2022	5.62	2.16	16.03	15.69
Daily	All	4.75	1.52	14.92	11.69
Monthly	2016	2.77	2.26	4.26	3.01
Monthly	2017	18.13	11.87	29.85	24.52
Monthly	2018	9.51	7.01	0.6	0.6
Monthly	2019	26.27	23.86	59.94	47
Monthly	2020	15.92	12.08	21.44	16.96
Monthly	2021	11.33	4.93	28.4	19.72
Monthly	2022	25.42	14.09	24.47	24.47

Table S6. Validation results for NicheMapR temperature (°C), using weather station data observed in Yale Myers Forest.

**Resolution**	**Year**	**Mean Temp RMSE**	**Mean Temp MAE**	**Min Temp RMSE**	**Min Temp MAE**	**Max Temp RMSE**	**Max Temp MAE**
Hourly	2017	4.29	3.21	NA	NA	NA	NA
Hourly	2018	4.04	3.19	NA	NA	NA	NA
Hourly	2019	3.99	3.11	NA	NA	NA	NA
Hourly	2020	3.85	3.03	NA	NA	NA	NA
Hourly	All	4	3.11	NA	NA	NA	NA
Daily	2017	2.76	2.12	3.03	2.5	3.1	2.31
Daily	2018	2.5	1.99	3.4	2.88	2.73	1.99
Daily	2019	2.38	1.83	3.4	2.87	2.51	1.92
Daily	2020	2.2	1.67	3.6	3.14	2.63	2.01
Daily	All	2.46	1.9	3.36	2.85	2.74	2.06
Monthly	2017	2.73	2.43	17.45	0.78	10.33	2.68
Monthly	2018	1.96	1.86	16.99	3.15	11.48	1.12
Monthly	2019	1.82	1.44	16.03	2.92	12.34	0.78
Monthly	2020	1.71	1.29	16.42	3.57	13.81	1.49
Monthly	All	2.06	1.75	16.72	2.61	11.99	1.52

Sources cited only in the Appendix

Abatzoglou, J. T. 2013. Development of gridded surface meteorological data for ecological applications and modelling. International Journal of Climatology 33:121–131.

Auguie, B., and A. Antonov. 2017, September 9. gridExtra: Miscellaneous Functions for “Grid” Graphics.

Bates, D., M. Mächler, B. Bolker, and S. Walker. 2015. Fitting Linear Mixed-Effects Models Using lme4. Journal of Statistical Software 67:1–48.

Billet, L. S., Y. A. Alshwairikh, L. K. Freidenburg, A. Rubinstein, S. Tracy, S. Nelson, and D. K. Skelly. 2024. Long-Term Decline of the Spotted Salamander (Ambystoma maculatum) in an Undeveloped Landscape. Herpetologica 80:11–21.

Campbell, G. S. 1985. Soil Physics with BASIC: Transport Models for Soil-Plant Systems. Elsevier.

Cheng, C.-T., M.-F. Chuang, T. Haramura, C.-B. Cheng, Y. I. Kim, A. Borzée, C.-S. Wu, et al. 2022. Open habitats increase vulnerability of amphibian tadpoles to climate warming across latitude. Global Ecology and Biogeography 32:83–94.

Costanzo, J. P., M. C. F. do Amaral, A. J. Rosendale, and R. E. Lee Jr. 2013. Hibernation physiology, freezing adaptation and extreme freeze tolerance in a northern population of the wood frog. Journal of Experimental Biology 216:3461–3473.

CT ECO. 2016. 2016 Lidar Elevation. Capitol Region Council of Governments.

Daniel, F., M. Corporation, S. Weston, and D. Tenenbaum. 2022, February 7. doParallel: Foreach Parallel Adaptor for the “parallel” Package.

Enriquez-Urzelai, U., M. R. Kearney, A. G. Nicieza, and R. Tingley. 2019. Integrating mechanistic and correlative niche models to unravel range-limiting processes in a temperate amphibian. Global Change Biology 25:2633–2647.

Groff, L. A., A. J. K. Calhoun, and C. S. Loftin. 2016. Hibernal Habitat Selection by Wood Frogs (Lithobates sylvaticus) in a Northern New England Montane Landscape. Journal of Herpetology 50:559–569.

Halverson, M. A., D. K. Skelly, J. M. Kiesecker, and L. K. Freidenburg. 2003. Forest mediated light regime linked to amphibian distribution and performance. Oecologia 134:360–364.

Hijmans, R. J., R. Bivand, E. Pebesma, and M. D. Sumner. 2022, December 2. terra: Spatial Data

Analysis.

Hollister, J., T. Shah, A. L. Robitaille, M. W. Beck, and M. Johnson. 2022, January 7. elevatr: Access Elevation Data from Various APIs.

Homer, C. G., J. A. Fry, and C. A. Barnes. 2012. The National Land Cover Database. Page Fact Sheet. U.S. Geological Survey.

Kearney, M. R., and W. P. Porter. 2017. NicheMapR – an R package for biophysical modelling: the microclimate model. Ecography 40:664–674.

Kearney, M. R., and W. P. Porter. 2020. NicheMapR – an R package for biophysical modelling: the ectotherm and Dynamic Energy Budget models. Ecography 43:85–96.

Liaw, A., and M. Wiener. 2002. Classification and fegression by Random Forest. R News 2.3 2:18.

Maclean, I. M. D., and D. H. Klinges. 2021. Microclimc: A mechanistic model of above, below and within-canopy microclimate. Ecological Modelling 451:109567.

Maclean, I. M. D., J. R. Mosedale, and J. J. Bennie. 2019. Microclima: An r package for modelling meso- and microclimate. Methods in Ecology and Evolution 10:280–290.

Neuwirth, E. 2022, April 3. RColorBrewer: ColorBrewer Palettes.

O'Connor, J. H., and T. A. G. Rittenhouse. 2016. Snow cover and late fall movement influence wood frog survival during an unusually cold winter. Oecologia 181:635–644.

R Core Team. 2024. A Language and Environment for Statistical Computing. R Foundation for Statistical Computing, Vienna, Austria.

Sinclair, B. J., J. R. Stinziano, C. M. Williams, H. A. MacMillan, K. E. Marshall, and K. B. Storey. 2013. Real-time measurement of metabolic rate during freezing and thawing of the wood frog, Rana sylvatica: implications for overwinter energy use. Journal of Experimental Biology 216:292–302.

Skelly, D. K., and L. K. Freidenburg. 2000. Effects of beaver on the thermal biology of an amphibian. Ecology Letters 3:483–486.

Tracy, C. R., K. A. Christian, and C. R. Tracy. 2010. Not just small, wet, and cold: effects of body size and skin resistance on thermoregulation and arboreality of frogs. Ecology 91:1477–1484.

Wickham, H. 2017. tidyverse: Easily Install and Load the "Tidyverse."

Wickham, H., and RStudio. 2021, November 26. conflicted: An Alternative Conflict Resolution

Strategy.

Zeileis, A., G. Grothendieck, J. A. Ryan, J. M. Ulrich, and F. Andrews. 2022, September 17. zoo: S3 Infrastructure for Regular and Irregular Time Series (Z's Ordered Observations).
